# Role of the cytoskeleton in cellular reprogramming: effects of biophysical and biochemical factors

**DOI:** 10.3389/fmolb.2025.1538806

**Published:** 2025-03-07

**Authors:** Ekaterina Momotyuk, Nour Ebrahim, Ksenia Shakirova, Erdem Dashinimaev

**Affiliations:** Center for Precision Genome Editing and Genetic Technologies for Biomedicine, Pirogov Russian National Research Medical University, Moscow, Russia

**Keywords:** cytoskeleton, cellular reprogramming, cell fate, mechanotransduction, chromatin, signal pathways, regenerative medicine

## Abstract

The cytoskeleton plays a crucial role in regulating cellular behavior, acting as both a structural framework and a mediator of mechanical and biochemical signals that influence cell fate. In the context of cellular reprogramming, modifications to the cytoskeleton can have profound effects on lineage commitment and differentiation efficiency. This review explores the impact of mechanical forces such as substrate stiffness, topography, extracellular fluid viscosity, and cell seeding density on cytoskeletal organization and mechanotransduction pathways, including Rho/ROCK and YAP/TAZ signaling. Additionally, we examine the influence of biochemical agents that modulate cytoskeletal dynamics, such as actin and microtubule polymerization inhibitors, and their effects on stem cell differentiation. By understanding how cytoskeletal remodeling governs cellular identity, this review highlights potential strategies for improving reprogramming efficiency and directing cell fate by manipulating mechanical and biochemical cues.

## Introduction

Cellular reprogramming refers to converting cell fate from one lineage to another by the forced expression of transcription factors and non-coding RNAs or through the effect of small molecules (Takahashi and Yamanaka, 2006). This process has garnered a lot of interest considering its potential to generate functional cells for therapeutic applications and has greatly reshaped our traditional views on cell identity and cell fate determination (Wang et al., 2021). Ongoing studies continually challenge the factors and methodologies that can alter cell identity, which in turn draws attention to the cytoskeleton as a crucial component of cellular identity and function.

The cytoskeleton is a network of dynamic filaments present in all cells that extends from the cytoplasm to the nucleus and supports diverse cellular functions in many cellular compartments. The distinctive components of the cytoskeleton in eukaryotic cells are actin filaments, intermediate filaments (IFs), and microtubules (Pollard and Goldman, 2018). These filaments are important to the functionality of cells, serving as mechanical support for the cytoplasm and cell surface (Pegoraro et al., 2017), providing tracks for molecular motors in the cells, which is crucial for the characteristic distributions of the cellular organelles (Xiao et al., 2016), cellular division (McIntosh, 2016), short-range movements of cellular vesicles (Titus, 2018) and signal transduction (Moujaber and Stochaj, 2020). For many years, cytoskeletal proteins were believed to be confined to the cytoplasms but later it was discovered that cytoskeletal proteins are associated with many nuclear processes. Inside the nucleus, the nucleoskeleton forms a dense filamentous network that encapsulates the genome; this nucleoskeleton is involved in maintaining nuclear structure and participates in cellular signaling (Dahl and Kalinowski, 2011).

Actin is one of the most fundamental proteins in eukaryotic cells comprising up to 20% of the total protein mass in some cell types (Klages-Mundt et al., 2018). Actin plays an integral part in maintaining cellular homeostasis taking part in gene activity, nuclear structure, and cellular reprogramming (Misu et al., 2017). It closely associates with RNA polymerases and regulates transcription at multiple levels (Fomproix and Percipalle, 2004; Kukalev et al., 2005; Xie and Percipalle, 2018). Actin-binding proteins (ABPs) have also been shown to localize to the cell nucleus, and are required for transcription elongation in an actin-dependent manner (Obrdlik and Percipalle, 2011). Other cytoskeletal proteins, like myosin (myo1c), also commonly known as nuclear myosin 1 (NM1) is found in the nucleus to different extents, and under different conditions participates with actin in transcription activation (Almuzzaini et al., 2015).

Considering the pivotal role of the cytoskeleton in many cellular processes, influencing the cytoskeleton with mechanical or biological cues has been shown to affect cellular functions and direct differentiation pathways, much like the influence of epigenetic or genetic factors (Wang et al., 2022). In this review, we aim to highlight the important role of the cytoskeleton in determining cell fate, its effects on signal transduction, and epigenetic regulation. Furthermore, the role of the cytoskeleton as a key mediator of mechanotransduction, translating mechanical forces, for example, substrate stiffness, compression, stretching, and biochemical signals targeting actin or microtubule polymerization in controlling cell fate and lineage commitments. This highlights the importance of cytoskeletal remodeling in regulating cellular identity and the potential to improve reprogramming outcomes by controlling mechanical and biochemical signals.

## The cytoskeleton and its components

**FIGURE 1:**
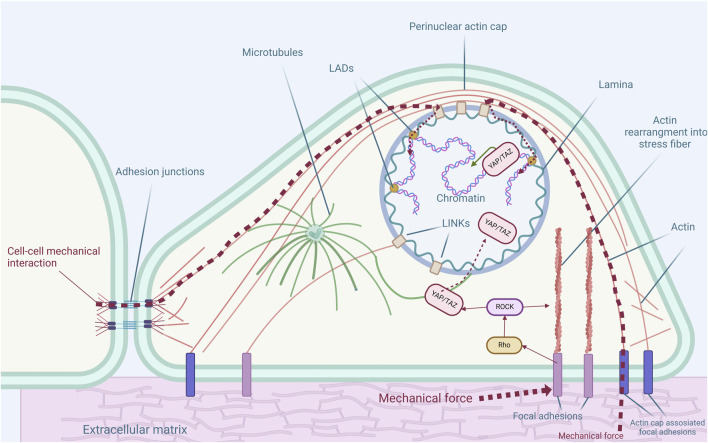
Cytoskeletal structures and their connections to nuclear and extracellular environment. The cytoskeleton is integral in maintaining cellular architecture and transmitting mechanical signals, impacting gene regulation and cell fate determination. Actin filaments maintain cellular shape, receive external mechanical signals by focal adhesions or adhesion junctions, and transmit them to the nucleus. The perinuclear actin cap encircles the nucleus, influencing the nuclear shape and gene expression. Microtubules extend from the microtubule organizing center (MTOC), maintain cell structure, and facilitate intracellular molecular transport. The nuclear lamina, along with lamina-associated domains (LADs), supports nuclear organization and chromatin positioning, while other intermediate filaments provide numerous different functions in the cytoplasm. Mechanical forces affect Rho/ROCK signaling which in turn affects YAP nuclear localization and further genomic events.

Although the term cytoskeleton traditionally suggests a role primarily in maintaining cell shape and facilitating motility, research over the past 30 years has revealed that the cytoskeleton is involved in a much broader range of cellular processes such as intracellular transport, cell movement, cell division, adhesion, reaction to external conditions, endocytosis, chromatin positioning, epigenetic regulation, and even direct participation in gene transcription. This multifunctionality is provided by a diversity of cytoskeleton components and their regulators. The three main components of the cytoskeleton are actin filaments, microtubules, and intermediate filaments (Figure 1). They are well-described in numerous reviews (Fletcher and Mullins, 2010; Hohmann and Dehghani, 2019), so we will give a brief overview of these key components, focusing on their impact on gene expression and cellular differentiation.

### Actin filaments

Actin filaments, also known as microfilaments, are critical components of the cytoskeleton, involved in various cellular processes such as motility, cell shape maintenance, mechanosensation, interactions with the extracellular matrix (ECM), and other cells via cell-cell adhesions.

Actin filaments are dynamic, filamentous structures formed by polymerizing monomeric globular actin (G-actin) into filamentous actin (F-actin). The dynamic reorganization of actin filaments is essential for proper cellular responses to both intracellular and extracellular signals. This reorganization is heavily dependent on ABPs, which regulate all aspects of filament organization. ABPs control filament assembly (profilin, actin Related Protein 2/3 complex (Arp2/3)), polymerization (formin, vasodilator-Stimulated Phosphoprotein (VASP)), depolymerization (actin-depolymerizing factor (ADF)/cofilin, gelsolin), capping (CP, tropomodulin), cross-linking (scruin, fascin), and branching (Arp2/3) (Pollard, 2016).

Actin filaments are not static, they rapidly polymerize on one end (the plus end), while on the other end (the minus end) a slower polymerization process is accompanied by filament disassembly to a monomeric state. This polarity arises from actin’s ATPase activity. G-actin is a weak ATPase, but its polymerization induces a conformational change that enhances ATP hydrolysis significantly (Guan et al., 2003). ATP-bound F-actin gradually converts to ADP-bound F-actin, with subsequent phosphate release (Blanchoin and Pollard, 2002). ADP-bound F-actin has a high affinity for the ADF/cofilin complex, which promotes filament disassembly (Carlier et al., 1997). Newly formed ends of microfilaments are called barbed; they are hotspots for the majority of the biochemical reactions and can either undergo further elongation or be stabilized by capping proteins that limit subunit addition or dissociation.

Stress fibers represent another important actin-based structure, consisting of contractile bundles of F-actin and myosin II, cross-linked by α-actinin (Naumanen et al., 2008; Sjöblom et al., 2008). These fibers are typically connected to focal adhesions (FAs) and play a crucial role in mechanotransduction - the process by which cells convert mechanical stimuli into biological responses (Blanchoin et al., 2014). FAs are macromolecular multiprotein sites where a cell connects with the ECM through binding between clustered transmembrane adhesion molecules (integrins) and specific FA proteins. FAs are essential for cell adhesion, mechanosensation, and translating mechanical forces on actin fibers into motile forces that drive cell migration (Wang et al., 2001; Legerstee and Houtsmuller, 2021).

Another actin structure that affects nucleus shape and cell fate determination is the perinuclear actin cap (not to be confused with the protein cap structure on the actin barbed ends). The perinuclear actin cap is a highly organized network of thick acto-myosin bundles covering the apical surface of the nucleus in adherent cells (Khatau et al., 2009). This structure is unique due to its direct connections with both the cell periphery and the nuclear scaffold via actin-cap associated focal adhesions (ACAFAs) (Kim et al., 2012) and Linker of Nucleoskeleton and Cytoskeleton (LINC) protein supercomplex (Crisp et al., 2006; Sgarzi et al., 2023) respectively. This organization allows actin cap to facilitate the transmission of forces from the extracellular matrix to the nucleus, influencing nuclear shape, chromatin organization, and tension-dependent signaling pathways, such as YAP/TAZ (Yes-associated protein/transcriptional co-activator with PDZ-binding motif) (Dupont et al., 2011). Sgarzi et al. (2023) demonstrated that in cancer cells with aberrant activation of hepatocyte growth factor receptor (HGFR), the actin cap is disrupted leading to nuclear shape irregularities and impaired cell motility. This disruption correlates with the relocation of YAP1, a key mechanotransducer, from the nucleus to the cytoplasm, resulting in its inactivation. The study demonstrates that HGFR ablation restores proper actin cap formation, YAP1 nuclear localization, and directional cell movement, underscoring the importance of the HGFR-YAP1 axis in regulating cytoskeletal organization and cell motility.

For many years, the presence and function of actin within the nucleus were subjects of skepticism. However, accumulating evidence now underscores the critical importance of nuclear actin (Kelpsch and Tootle, 2018; Venit et al., 2018). Multiple studies have demonstrated that actin is essential for transcriptional machinery through its interactions with RNA Polymerase (RNAP) I (Philimonenko et al., 2004), RNAPII (Hofmann et al., 2004; Zhu et al., 2004), and RNAPIII (Hu et al., 2004). Moreover, actin acts as a regulator of gene expression in response to environmental changes. A recent study revealed that nuclear actin, in conjunction with the actin-binding protein complex Wiskott–Aldrich syndrome protein (N-WASP)/Arp2/3, induces a serum-dependent transcriptional program by scaffolding active and long-lasting RNAPII under serum stimuli (Wei et al., 2020).

Over the past decade, more examples of the role of actin in gene regulation have emerged. A significant amount of data comes from studies on mesenchymal stromal/stem cells (MSCs). While MSCs have the potential to differentiate into various lineages, the balance between osteogenic and adipogenic differentiation represents a particularly well-characterized dichotomy. These studies have highlighted how actin dynamics and cytoskeletal organization can influence the decision between these two fates (Figure 1). For example, it was shown that in MSCs the persistent long-term presence of intranuclear actin induces the Runx2-dependent expression of osteogenic genes such as osterix (*Osx*) and osteocalcin (*Ocn*), leading to the acquisition of an osteogenic cell phenotype (Sen et al., 2015). The Arp2/3 complex, which facilitates actin filament branching, is crucial in this process; its absence strongly promotes adipogenesis rather than osteogenesis (Sen et al., 2017; Sen et al., 2024). Another key actin-binding protein essential for genome organization is the nuclear-localized formin diaphanous-related formin 3 (mDia2). mDia2 plays a crucial role in maintaining the integrity of the nuclear actin-lamin structure. Loss of mDia2 disrupts the lamin B1 structure at the nuclear envelope, compromising the actin-lamin nucleoskeleton and subsequently triggering Runx2-dependent osteogenic differentiation in MSCs (Sankaran et al., 2020).

### Microtubules

Microtubules are the largest and most rigid components of the cytoskeleton. Similar to actin, they play a crucial role in maintaining cell shape and other various cellular processes, including cell motility, cell division, intracellular transport, and cellular signaling.

Structurally, microtubules are cylindrical hollow polymers composed of approximately 13 linear protofilaments (PFs) formed by α- and β-tubulin dimers (Goodson and Jonasson, 2018). Among the various tubulin isoforms identified, γ-tubulin is particularly significant due to its critical role in nucleating new microtubule structures (Sulimenko et al., 2022). γ-tubulin is highly concentrated in the microtubule organizing center (MTOC) - a cellular region where new microtubules are generated. Centrosomal MTOC plays a major role in cell division.

Similar to actin filaments, microtubes are polarized and dynamic structures. Their assembly dynamics closely resemble those of actin filaments, although microtubules utilize GTP hydrolysis rather than ATP (Hyman et al., 1992). GTP-bound tubulin heterodimers are added to the plus end of the growing microtubule, where they subsequently hydrolyze GTP to GDP, facilitating further depolymerization (Goodson and Jonasson, 2018). The assembly of microtubules is initiated at the MTOC, anchoring the minus ends within the MTOC and orienting the plus ends toward the cell periphery.

Cycles of de/polymerization are frequently interrupted by the sudden switch from growing to quick disassembly, followed by a new growth cycle. This behavior is called dynamic instability and it is believed to enable microtubule tips to efficiently explore cellular space, enhancing their ability to locate and interact with specific targets within the cell (Gudimchuk and McIntosh, 2021).

Microtubules play a fundamental role in intracellular transport, facilitating the movement of organelles, vesicles, and macromolecules within the cell. This transport function is mediated primarily through the interaction of microtubules with motor proteins, such as kinesins and dyneins, which convert chemical energy into mechanical work, enabling the directional movement of cargo along the microtubule tracks. Kinesins generally move cargo toward the plus end of microtubules to the cell periphery while dyneins transport cargo toward the minus end to the MTOC (Hirokawa et al., 2009). Microtubule-associated proteins (MAPs) contribute to the regulation of microtubule-based transport. These proteins can stabilize microtubules, regulate their interactions with motor proteins, and coordinate cargo attachment, thus modulating transport efficiency and specificity (Mandelkow and Mandelkow, 2002).

Aside from the orientation of the mitotic spindle, microtubules' role in cell fate also lies in mechanotransduction, which will be discussed later in the article, and regulation of such important developmental signaling pathways as Wnt. It was shown that dynein interacts with β-catenin, a protein that acts as a transcriptional co-activator in Wnt signaling. Disruption of microtubule dynamics results in improper β-catenin localization, reducing its nuclear translocation and subsequent transcriptional activation of Wnt target genes (Ligon et al., 2001). This regulation is essential in stem cell differentiation and tumorigenesis.

Another pathway through which microtubules affect cell differentiation is Hippo. The Hippo pathway is a key regulator of organ growth, cell proliferation and differentiation, embryogenesis, and tissue regeneration/wound healing, operating through the activity of YAP/TAZ (Fu et al., 2022). Microtubules play a significant role in regulating the subcellular localization of YAP/TAZ: when microtubules are destabilized, YAP/TAZ tends to localize in the cytoplasm, where it becomes inactive, preventing transcriptional activation of its target genes, and conversely, stable microtubules promote YAP/TAZ nuclear translocation, where they activate genes associated with cell proliferation, survival, and stem cell maintenance (Dupont et al., 2011).

### Intermediate filaments

IFs differ from actin filaments and microtubules in their structural diversity and functional roles. Regardless of their type, IFs are composed of proteins that form filaments with a uniform diameter of approximately 10 nm and exhibit an organized α-helical conformation, which favors the formation of two-stranded coiled coils, contributing to the greater flexibility and mechanical strength of IFs (Herrmann and Aebi, 2016).

Intermediate filaments are classified into six types based on sequence homology (Szeverenyi et al., 2008).

• Type I and II are acidic and basic keratins, respectively. They are predominantly found in epithelial cells and form heteropolymeric filaments essential for the structural integrity of the epidermis and its appendages.
• Type III includes four homopolymer-forming proteins—vimentin, desmin, peripherin, and glial fibrillary acidic protein (GFAP). Vimentin is widely expressed in mesenchymal cells, desmin is found in muscle cells, peripherin is present in peripheral neurons, and GFAP is specific to astrocytes and other glial cells.
• Type IV contains neurofilament heteropolymers: NF-L, NF-M, NF-H (neurofilament light, medium, and heavy, respectively); internexin and synemin.
• Type V proteins are lamins, a major component of the nuclear envelope. Lamins produce nuclear lamina - a dense protein network under the inner nuclear membrane. Lamina gives mechanical stability to the nucleus, providing structural protection and organization for DNA. Mutations in the lamins gene *LMNA* are known to cause diseases, termed laminopathies, genomic instability, and malignancy (Liu et al., 2005). There are different types of lamins with affinity to different-state chromatin: lamin A/C is predominantly associated with euchromatic regions, whereas lamin B is primarily linked to heterochromatin (Gesson et al., 2016). Lamins play an important role in epigenetic regulation of gene expression, which will be described in the next section.
• Type VI IFs are also known as beaded filaments, they are characterized by their distinctive beaded morphology. VI type includes nestin, tanabin, synemin, and transitin (Guérette et al., 2007).

In contrast to the dynamic nature of actin filaments, the lamin A/C nucleoskeleton appears relatively static in fully differentiated cells until deregulated by aging, cancer, or epithelial-to-mesenchymal transition (EMT) (Heo et al., 2016; Sankaran et al., 2020; Pang et al., 2024). However, subtler rearrangements still occur in response to various stimuli, such as mechanotransduction. The nuclear lamina plays a key role in transmitting biomechanical forces to the cell nucleus and chromatin. It is anchored to the cytoskeleton through a nuclear envelope protein complex called LINC (linker of nucleoskeleton and cytoskeleton) (Mellad et al., 2011). The LINC complex is composed of two families of integral membrane proteins: SUN (Sad1p, UNC-84) and conserved C-terminal KASH (Klarsicht/ANC-1/Syne Homology) proteins. SUN proteins are located in the inner nuclear membrane, where they interact with lamins. They extend into the perinuclear space, where they interact with KASH proteins, which are anchored in the outer nuclear membrane. The KASH proteins then extend into the cytoplasm, where they engage with the cytoskeleton (Bouzid et al., 2019; King, 2023). Lamins, especially lamin A, are critical mediators in mechanotransduction; the integrity of the lamina affects how the nucleus and the cell respond to mechanical stress, such as shear stress or substrate stiffness (Donnaloja et al., 2020; Sapra et al., 2020). Indeed, matrix stiffness directly influences lamin A protein levels: stiff substrates increase lamin A levels, leading to osteogenic differentiation of stem cells, whereas soft matrices are associated with low lamin A levels and adipogenic differentiation (Engler et al., 2006; Swift et al., 2013). These findings correlate with results from experiments involving knockdown and overexpression of the lamin A gene, *LMAC*: LMAC deficiency promotes adipocyte differentiation, while overexpression of *LMAC* increases osteogenic differentiation (Scaffidi and Misteli, 2008; Akter et al., 2009; Swift et al., 2013; Tsimbouri et al., 2014).

### Cytoskeleton role in epigenetic regulation

Chromatin structure plays a crucial role in key cellular processes by regulating accessibility to DNA, thereby influencing the interactions of proteins and other factors that are essential for development and differentiation. Chromatin-modifying complexes are responsible for activating and repressing transcription at specific chromosomal regions through epigenetic modifications (Klages-Mundt et al., 2018). These complexes can be categorized into subfamilies based on their central ATPase components. Among them, SWItch/Sucrose Non-Fermentable (SWI/SNF) complexes are particularly significant due to their involvement in various processes, including transcriptional regulation and the modulation of genes associated with cell adhesion and ECM proteins (Alfert et al., 2019). Furthermore, SWI/SNF complexes containing Brahma-related gene 1 (BRG1) and BRAHMA (BRM) ATPase units are critical for regulating the expression of genes necessary for cellular proliferation and differentiation, establishing their close association with cell cycle regulation (Hogan and Varga-Weisz, 2007).

There is substantial evidence indicating a correlation between chromatin-modifying complexes and cytoskeletal proteins, particularly nuclear actin. Β-actin is a ubiquitously expressed isoform of actin found in the nucleus, where it plays several significant roles, particularly in shaping the chromatin landscape (Dugina et al., 2022). Actin and ARPs play crucial roles in the assembly and regulation of chromatin-modifying complexes (Klages-Mundt et al., 2018), facilitating transitions between transcriptionally active chromatin compartments (A-compartments) and repressed chromatin compartments (B-compartments), which correspond to increased and decreased gene expression, respectively. Notably, several key regulators of cell differentiation, such as SRY-Box Transcription Factor 21 (SOX21), Bone morphogenetic protein 3 (BMP-3), and BMP-6, are among the genes influenced by these chromatin landscape changes (Sen et al., 2024). Actin-binding proteins are essential in the recruitment, assembly, and maintenance of the structural integrity of chromatin remodeling complexes (Pollard, 2016). The relationship between ARPs and chromatin-modifying complexes was thoroughly reviewed by (Pollard, 2016). ARPs can form heterodimers with each other, such as ARP7-ARP9, or pair with actin, as in the Actin-ARP4 complex, to promote the structural integrity of chromatin-modifying complexes (Clapier and Cairns, 2009; Schubert et al., 2013). Among these, ARP4 is the most commonly identified ARP in chromatin-modifying complexes, where the Actin-ARP4 pair interacts with the HSA domain unique to each complex (Farrants, 2008).

Actin and ARPs also affect chromatin regulation independently of their physical involvement in chromatin-modifying complexes by directly interacting with histones (Klages-Mundt et al., 2018). The absence of β-actin leads to the downregulation of epigenetic marks for active chromatin such as acetylation on lysine 9 of histone H3 (H3K9ac) and trimethylation of lysine 4 on histone H3 (H3K4me3) at rDNA loci. While epigenetic marks for repressive chromatin such as monomethylation of lysine 4 on histone H3 (H3K4me1) were found to be upregulated (Almuzzaini et al., 2016). Moreover, knockout of β-actin in mouse embryonic fibroblasts leads to alteration of the heterochromatin landscape across the genome compared to wild-type cells, specifically accompanied by increased methylation of histone 3 (H3K9Me3) levels in the majority of chromatin regions (Xie et al., 2018a). This increased H3K9me3 methylation is also implicated in the induction of neural gene programs. Directly reprogrammed β-actin knockout embryonic fibroblasts into neurons contain increased levels of H3K9Me3 along with loss of the ATPase subunit of the chromatin-modifying complex BAF at transcription start sites of multiple gene loci (Xie et al., 2018b). Reduction of cytoskeletal tension using chemical agents like blebbistatin in primary fibroblasts being differentiated into neurons downregulated the expression of heterochromatin genes manifested by the decreased marks H3K27me3 and H3K9me3, while promoting an open chromatin structure globally and locally, manifested by an increase in AcH3, H3K4me3, and H3K4me1 marks. Furthermore, Blebbistatin treatment increased histone acetyltransferase (HAT) and H3K4-specific histone methyltransferase (HMT) activity while reducing histone deacetylase (HDAC) and histone demethylase (HDM) activity. This could lead to increased histone H3 acetylation and H3K4 methylation, promoting gene activation. Additionally, it increased accessibility at the promoter or enhancer regions of neuronal genes (Soto et al., 2023).

Besides actin, Nuclear myosin 1 participates in the recruitment of histone acetyltransferases (HATs) and histone methyltransferases (HMTs) which promote an epigenetic landscape compatible with active transcription (Almuzzaini et al., 2015). NM1 was also found to be a part of chromatin modifying complex B-WICH which interacts with actin forming an actomyosin molecular motor affecting the attachment of RNA polymerase 1 with the chromatin (Ye et al., 2008).

Furthermore, studies have found the cell geometry to affect the nuclear-cytoplasmic relocalization of SET And MYND Domain Containing 3 (SMYD3) lysine methyltransferase in murine myoblasts. This distribution in response to cell geometry was correlated with cytoplasmic and nuclear lysine tri-methylation levels and it could change SMYD3 substrates and subsequent nuclear vs. cytoplasmic functions (Pereira et al., 2020). Cell geometry has also been found to affect cytoplasmic-to-nuclear redistribution of histone deacetylase 3 in an actomyosin-dependent manner which in turn affects chromatin compaction (Jain et al., 2013). Another study demonstrated that applying cyclic stretch to fibroblasts during reprogramming into induced pluripotent stem cells (iPSCs) significantly boosted reprogramming efficiency. The stretched cells showed epigenetic modifications, particularly a reduction in H3K9me3, along with global and gene-specific changes in chromatin occupancy, which contributed to the improved generation of iPSCs (Park et al., 2023).

Chromatin organization and maintenance also rely on lamins: lamin A/C is predominantly associated with euchromatic regions, whereas lamin B is primarily linked to heterochromatin (Gesson et al., 2016). Maintaining the nuclear organization depends on several components, one of which is nuclear lamina and specialized topologically associating domains named lamina-associated domains (LADs) (van Steensel and Belmont, 2017; Rowley and Corces, 2018). This organization is vital for regulating gene expression by controlling the accessibility of DNA to transcription factors and other regulatory proteins.

Although LADs often interact with the nuclear lamina, these two entities are distinct and should not be confused. The nuclear lamina is a network underlying the nuclear envelope, while LADs are heterochromatin regions located at the nuclear periphery, characterized as transcriptionally repressed and enriched with repressive histones such as H3K9me2, H3K9me3, and H3K27me3 (van Steensel and Belmont, 2017). LADs are distributed along heterochromatin on chromosome arms but are not associated with pericentromeric heterochromatin (Guelen et al., 2008; Peric-Hupkes et al., 2010; Ho et al., 2014).

The molecular mechanisms underlying the transcriptional regulation of LAD are not completely discovered yet, but there is strong evidence that at least one of the mechanisms is dependent on dynamic binding to the nuclear lamina, however not limited by it due to weak correlation between nuclear lamina loosening and changes in gene expression that was shown in several recent studies (Forsberg et al., 2019; Chang et al., 2022).

Regulators of lamina binding can be broadly classified into two categories: tethers and looseners (Manzo et al., 2022). Lamins themselves, particularly lamin B and C, play a significant role in anchoring LADs to the lamina, but they are not the only regulators involved (Ulianov et al., 2019; Wong et al., 2021; Chang et al., 2022). Lamin B Receptor (LBR) appears to be one of the most crucial tethers in mammals. Studies have shown that knocking out or down-regulating LBR disrupts the organization of LADs, leading to abnormal chromatin remodeling and gene expression (Solovei et al., 2013; Falk et al., 2019; Herman et al., 2021; Schep et al., 2021). Notably, LBR deficiency is strongly associated with cellular senescence (Arai et al., 2019; En et al., 2020), highlighting the importance of proper chromatin organization for cellular health and suggesting that LBR’s role in suppressing genome instability could make it a potential target for promoting cellular longevity.

Several other proteins have been identified as potential tethers of LADs, including chromo domain-containing protein Ces-4 in *Caenorhabditis elegans* (Bian et al., 2020) and proline-rich 14 (PRR14) (Poleshko et al., 2013; Dunlevy et al., 2020), PR/SET Domain 16 (Prdm16) (Biferali et al., 2021), and Zinc Finger With KRAB And SCAN Domains 3 (ZKSCAN3) (Hu et al., 2020) in mammals. The exact mechanisms by which these proteins tether LADs remain to be fully elucidated, though evidence suggests that these mechanisms may involve the recognition of H3K9me marks on heterochromatin (Poleshko et al., 2019; Bian et al., 2020; Biferali et al., 2021).

Specific looseners of LADs have yet to be identified, but it has been shown that forced gene activation within a LAD can cause local detachment of chromatin from the nuclear lamina, affecting around 50 kb flanking the activated site, and *vice versa* (Brueckner et al., 2020; Su et al., 2020). Histone acetylation is another factor that appears to weaken chromatin-nuclear lamina interactions. For example, in *C. elegans*, the loss of the euchromatin binder Mrg1, which normally sequesters histone acetyltransferases, results in increased histone acetylation and subsequent LAD detachment (Cabianca et al., 2019). Similarly, in mammalian cells, depletion of histone deacetylase SIRT3 increases accessibility in LADs, possibly due to increased histone acetylation (Diao et al., 2021).

### Cytoskeleton rearrangement in mature cells during EMT

In understanding the role of the cytoskeleton in cell fate alteration it is important to observe natural cases of mature cytoskeleton rearrangement. Here we will describe an epithelial-to-mesenchymal transition (EMT) - a process that is based on such significant morphological alterations as malignancy and re-epithelialization during wound healing.

EMT is a biological process during which apical-basal polar epithelial cells undergo multiple transcriptional, biochemical, and morphological changes that enable them to weaken strong cell-to-cell junctions, detach from the basal membrane, and obtain a back–front polar mesenchymal cell phenotype, which enhances cells’ migratory capacity due to their dynamic attachment to the extracellular matrix, invasiveness, elevated resistance to apoptosis, and increased production of ECM components (Kalluri and Weinberg, 2009). EMT occurs normally during early embryonic development as well as during wound healing in adults, cancer pathogenesis, and tissue fibrosis. It is important to note that the transition from an epithelial to a mesenchymal phenotype is often incomplete, resulting in cells that occupy various intermediate states depending on their biological context (Nieto et al., 2016).

Cells undergo EMT in response to environmental factors that activate EMT-inducing signaling pathways. The most prominent and well-known pathways include the transforming growth factor-beta (TGFβ) and Wnt pathways, which trigger the expression of EMT-specific transcription factors (TFs) such as Snail, Slug, and Twist 1/2, among others (Díaz-López et al., 2014).

Although the dynamics of cytoskeletal changes during EMT have been a subject of considerable interest [reviewed in Datta et al. (2021)], some aspects, like cytoskeleton dynamics during EMT or the role of microtubules during EMT remain underexplored. Induced EMT stimulates a massive reorganization of actin into stress fibers and their alignment in both cancerous and non-cancerous cell lines (Nieto et al., 2016). These aligned thick actin fibers help form prominent protrusions at the leading edge, which are essential for cell mobility and migration (Datta et al., 2021).

Changes in FAs during EMT depend on cell type greatly. However, two morphological features are common across studied cell lines: a decrease in the average area of FAs and an increase in the frequency of FAs (Geiger et al., 2008; Bianchi et al., 2010; Nurmagambetova et al., 2023). There is a hypothesis suggesting a positive correlation between the size of FAs and cell speed, but the data remains inconclusive (Kim and Wirtz, 2013; Nurmagambetova et al., 2023).

The most significant changes during EMT occur in the organization of microtubules. The spatial organization of the microtubule array becomes more radial, especially at the cell edges. After EMT, cells display a larger area covered by microtubules, while their density at the cell edges decreases. This could indicate microtubule growth in the cell interior rather than at the periphery (Kiss et al., 2018; Nurmagambetova et al., 2023).

Although cytoskeletal remodeling is generally considered a downstream event in EMT, regulated by specific signaling cascades, several examples of the cytoskeleton directly regulating EMT have been documented. For instance, Kelch Like Family Member 23 (KLHL23), an inhibitor of actin polymerization, inversely suppresses EMT (Peng et al., 2018). Further investigation revealed that actin remodeling promotes EMT through the induction of hypoxia-inducible factor and Notch signaling in a cell-density-dependent manner. Actin remodeling also contributes to the disruption of E-cadherin at cell-cell adhesions, facilitating cell detachment from its microenvironment (Yilmaz and Christofori, 2009).

Pascual-Reguant and colleagues discovered a new type of LADs called euchromatin LADs (eLADs), which are formed by lamin B1 and euchromatin regions (Pascual-Reguant et al., 2018). eLADs are dynamic and change during TGF-β-induced EMT: as EMT begins, the amount of lamin B1 increases at TAD borders, strengthening these borders. Over time, additional eLADs form around transcriptionally active genes involved in the EMT pathway. Once cells acquire a mesenchymal phenotype, there is a tendency for these eLADs to become inactive. These findings suggest that lamin B1 might play a critical role as an architectural protein in establishing new genomic conformations and transcriptional patterns pivotal for new cell types during EMT (Pang et al., 2024).

Given that EMT represents a natural example of transdifferentiation, where cells undergo significant cytoskeletal rearrangements, signaling cascades, and changes in nuclear architecture, these processes offer valuable models for studying cellular plasticity. Leveraging factors and signaling pathways associated with EMT and cytoskeletal remodeling components as additional reprogramming agents can potentially drive transdifferentiation in controlled settings, thus facilitating new protocols for cell fate manipulation in regenerative medicine and tissue engineering.

## Biophysical and biochemical agents affect the cytoskeleton during cellular reprogramming

### Biophysical forces and the cytoskeleton

**FIGURE 2:**
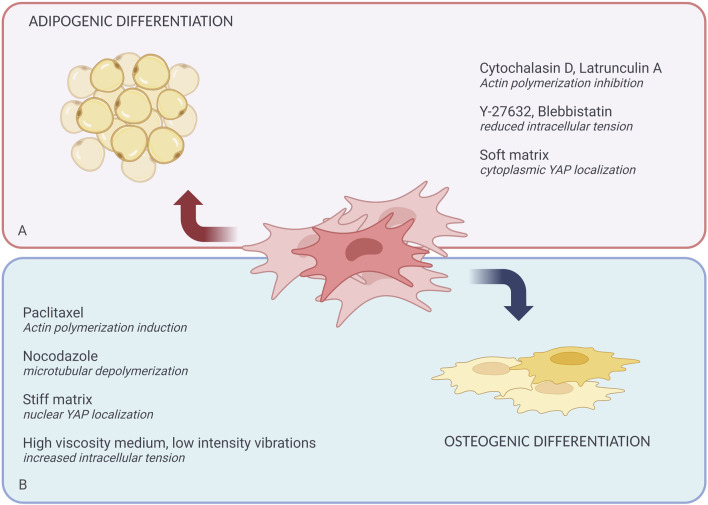
Role of the cytoskeleton and mechanotransduction in defining MSCs fate. Extrinsic biochemical agents and physical forces can affect MSCs differentiation. **(A)** Shows the chemical agents that promote adipogenic differentiation like Cytochalasin D, Lantrunculin A, Y-27632, Blebbistatin; and physical forces like soft matrix. **(B)** Shows the chemical agents that promote osteogenic differentiation including Paclitaxel, Nocodazole, and physical forces like stiff matrix, high viscosity media, and low-intensity vibrations.

The mechanical environment surrounding the cell, including the properties of the ECM and external forces such as tension, compression, and shear stress, significantly influence cellular behavior, including differentiation and identity switching (Engler et al., 2006; Melo-Fonseca et al., 2023) (Figure 2). The link between mechanical forces and gene expression is mediated by mechanosensitive proteins and signaling pathways in mechanotransduction, which involves the conversion of mechanical signals into biochemical signals via the cytoskeleton. FA complexes anchor the cytoskeleton to the ECM and play a key role in sensing mechanical cues. These complexes can activate a cascade of intracellular signaling pathways, including the Rho/ROCK and YAP/TAZ pathways (Dupont et al., 2011).

Rho signaling specifically facilitates actin polymerization and the formation of stress fibers, essential for maintaining cell shape and helping regulate the contractility required for cells to sense and respond to matrix stiffness and mechanical forces (Xie et al., 2023). Moreover, Rho/ROCK activation affects YAP localization (Nardone et al., 2017), which functions as a mechanosensor and translocates from the cytoplasm into the nucleus in response to mechanical forces (Elosegui-Artola et al., 2017) (Figure 1). Subsequently, the actin filament network is rearranged and aligned perpendicular to the direction of mechanical stress, as demonstrated in hMSCs under tensile stress (Parandakh et al., 2017).

As a result, mechanical pathways converge in the nucleus, where chromatin stretches and unfolds, influencing the transcriptional apparatus and further activation of gene expression (Tajik et al., 2016). During mechanical stimulation, the link between the nucleus and the cytoskeleton is mediated by the LINC complex (Crisp et al., 2006), which translates these signals into intracellular responses. This sensitivity is particularly pronounced concerning substrate stiffness, where LINC complexes promote mechanosensitive gene expression, suggesting that they are integral to the cellular perception of environmental mechanical cues (Alam et al., 2016).

During development, genetic programs and soluble morphogens regulate proliferation, differentiation, and tissue organization. However, mechanical forces impact numerous cell functions (Wozniak and Chen, 2009). For example, stiffness is important during embryogenesis, Cells sense stiffness through actomyosin-based contractility linked to integrin adhesions, which cluster in response to substrate stiffness, activating mechanosensitive proteins and downstream transcription factors (Janmey et al., 2020). *In vitro* studies use different kinds of substrates to study the interaction between cells and their environments, however, tissues and ECMs exhibit more complex mechanical behaviors, including viscoelasticity and non-linear elasticity which are critical during development and disease (Chaudhuri et al., 2020). For example, during *Xenopus laevis* gastrulation, mesoderm and notochord stiffness prevent buckling, while the involuting marginal zone stiffens to maintain structural integrity (Adams et al., 1990). Mechanical feedback also plays a crucial role in regulating proliferation; during *Drosophila melanogaster* oogenesis, localized myosin-generated tension drives epithelial cell proliferation to accommodate tissue growth, while reduced myosin activity suppresses proliferation and deforms tissues, suggesting tension promotes growth while compression slows it (Wang and Riechmann, 2007). Furthermore, in *D. melanogaster* gastrulation, endogenous tissue compression during germband extension (GBE) upregulates Twist expression, a key regulator of mesoderm and midgut differentiation (Desprat et al., 2008). Furthermore, the effect of mechanical stimulation can push cells into a malignant state, a recent study demonstrated that in a 3D breast cancer culture model, a stiff ECM induces a tumorigenic phenotype by altering the chromatin state. Increased ECM stiffness leads to cells with more wrinkled nuclei and increased lamina-associated chromatin. Cells grown on stiff matrices exhibited more accessible chromatin regions with Sp1-binding footprints. This transcription factor, in conjunction with histone deacetylases 3 and 8, plays a key role in regulating stiffness-induced tumorigenicity (Stowers et al., 2019). Tumor cells in turn progressively remodel cytoskeletal structures and reduce cellular stiffness during tumor progression, which makes targeting cytoskeletal components a target in controlling tumorigenic potential *in vivo*. Weakening/strengthening actin cytoskeleton can facilitate β-catenin nuclear/cytoplasmic localization, β-catenin in turn binds to the promoter of Oct4, activating it and sustaining self-renewal and malignancy (Chen et al., 2023). To further understand how the cellular environment affects cellular processes, we review in the following section some of the mechanical forces and their effects on cells *in vitro*.

#### ECM and topography

Сells cultured on substrates with varying stiffness can experience different extents of cytoskeletal tension, which in turn influences cell morphology and fate. For example, it has been observed that cells on stiff surfaces show enhanced FA maturation and a more organized actin cytoskeleton, facilitating greater cell spreading (Yeung et al., 2005). Conversely, exposure to softer ECM results in more rounded cell shapes, correlating with a relaxed cytoskeletal state, for example, reprogramming of MSCs by reducing intracellular tension on a substrate with low elastic modulus promotes phenotypic changes similar to pluripotent stem cells (Gerardo et al., 2019).

Depending on the dimension of the ECM, cell fate preferences and signal perception may differ. One of the examples is the differentiation of MSCs, which in 2D culture differentiates into adipocytes when cultured in elastic environments, while on stiffer substrates osteogenesis is promoted (Engler et al., 2006; Vilar et al., 2023). Transcription factors YAP and TAZ play important roles in the differentiation of MSCs into specific cell lineages, particularly under the influence of ECM stiffness. Studies reveal that MSCs lacking YAP/TAZ and cultured on rigid substrates do not successfully differentiate into osteogenic lineages (Dupont et al., 2011; Na et al., 2024). Instead, they preferentially undergo adipogenic differentiation, a response similar to what occurs in environments with softer substrate conditions. Interestingly, in 2D cultures, a soft matrix is required for adipogenic differentiation, whereas in 3D cultures a stiffer matrix is required to achieve the same result (Oliver-De La Cruz et al., 2019). In 2D cultures, nuclear YAP/TAZ localization tends to increase with increasing substrate stiffness, while in 3D environments, the relationship is more complex due to the influence of local stiffness and the spatial arrangement of the ECM (Caliari et al., 2016). This highlights the importance of dimensionality in influencing cell fate.

Actin cytoskeleton is influenced not only by substrate stiffness but also by its topography at the micro- or nanoscale (Lou et al., 2019; Belay et al., 2023). With the help of certain material geometries, it is possible to regulate the tension of the cytoskeleton, which can strengthen or weaken the connection with the ECM and possibly improve the process of cell reprogramming. As shown by Soto et al. (2023), the use of micro or nanomaterials reduced cell spreading and FA signaling, which facilitated the conversion of fibroblasts to induced neurons (Soto et al., 2023). Besides micro or nano levels of matrix topography, its patterns also matter. A study by Li et al. (2023), where MSCs were cultured on 3D micropatterns with various patterns, revealed that nuclear translocation of YAP was significantly higher on triangular prism and cuboid patterns compared to those on cylindrical and cubical patterns (Li et al., 2023). All these data underline the significance of matrix characteristics like stiffness and topography in responses to mechanical signals.

#### Extracellular fluid (ECF) viscosity

Another factor influencing cytoskeletal reorganization and signaling pathways, including YAP translocation is ECF viscosity. Viscosity influences integrin-dependent cell spreading and mechanotransduction, leading to the nuclear translocation of YAP and β-catenin (Gonzalez-Molina et al., 2018). These correlate with a recent paper (Chen et al., 2024) where Human MSCs cultured in high viscosity media showed larger cell spreading area and higher intracellular tension leading to increased formation of actin stress fibers and promoting nuclear localization of nuclear factor of activated T cells 1 and YAP, required for osteogenic gene expression. The effect of high viscosity on the actin cytoskeleton is explained by the activation of the ARP2/3 complex, which facilitates cell motility and contractility via the RhoA signaling pathway (Bera et al., 2022). During cell reprogramming, it is important to consider the synergistic effect of ECF and ECM. Studies have shown that increased ECF viscosity significantly enhances cellular mechanotransduction, particularly on rigid substrates (Cao et al., 2023).

#### Сell seeding density

Сell seeding density can significantly affect the mechanical stress experienced by cells. Seeding density affects the cytoskeleton by modulating cell adhesion and spreading characteristics. As the density of cells increases, their adhesion to the substrate diminishes, leading to reduced cell spreading, whereas cell-cell contacts and paracrine signaling become more prevalent (McBeath et al., 2004). This shift can alter the mechanical properties of the cytoskeleton, as cells under high density tend to experience greater confinement, affecting their shape and function within the tissue environment. Increased cell density leads to reduced expression of pluripotency genes and enhanced differentiation, as seen in human pluripotent stem cells (hPSCs) where high density diminishes YAP activity, crucial for maintaining pluripotency (Hsiao et al., 2016). Conversely, low cell density enhances differentiation efficiency in mouse embryonic stem cells (mESCs) by facilitating the nuclear translocation of β-catenin, which promotes lineage-specific gene expression (LeBlanc et al., 2022). Low cell density can indeed minimize mechanical stress experienced by individual cells, which may contribute to their survival and function during reprogramming (Kogut et al., 2018). However, in another study using a mechanical device, reprogramming efficiency was enhanced at high cell density (Sia et al., 2016), suggesting that conditions need to be selected for specific reprogramming.

#### Devices and manipulation

The use of various devices that can affect the reorganization of the cytoskeleton can be a good addition to reprogramming protocols. For example, horizontal low-intensity vibrations (LIV) application were effective in reorganizing the cytoskeleton of human bone marrow MSCs, which contributed to increased cell rigidity and upregulation of genes associated with matrix maturation, osteogenesis, and cytoskeletal organization (Pongkitwitoon et al., 2016). Cytoskeletal reorganization by LIV exposure activates RhoA mechanical signaling, as well as increases the formation of new FA and likely enhances nuclear-cytoskeletal coupling (Uzer et al., 2015).

Microfluidic devices have also been demonstrated to exert mechanical effects on the cytoskeleton. Using a microfluidic device to apply mechanical compression on fibroblasts during direct reprogramming into neurons has been shown to affect the reprogramming process. Millisecond compression resulted in transient nuclear deformation that influenced chromatin remodeling, increased programming efficiency, and the expression of the endogenous neuronal marker ASCL1 compared to controls (Song et al., 2022). Compression activates cytoskeletal reorganization (Schmitter et al., 2023) and activates RhoA and ROCK signaling (Boyle et al., 2020).

Among mechanical effects, shear stress *in vitro* can also be reproduced using microfluidic devices, which closely mimic the physiological conditions experienced by cells in blood vessels and other fluid environments. These systems allow precise control of fluid flow rates that impart shear stress to cells (Dash et al., 2020; Yu et al., 2024). Such application of shear stress has been shown to influence the direct reorganization of F-actin, gene expression patterns, and cellular signaling pathways (Kuo et al., 2015). For example, in smooth muscle cells, shear stress triggers cytoskeletal reorganization and epigenetic reprogramming through cofilin-dependent mechanisms, influencing integrin signaling and extracellular matrix remodeling (da Silva et al., 2019). Using MSCs as an example, it was shown that shear stress applied using an orbital shaker, in synergy with chemical inducers, promoted the differentiation of MSCs into endothelial cells, although it did not show such results when exposed to shear stress alone (Homayouni Moghadam et al., 2014). This shows that mechanical stress can be an additional inducer to increase the efficiency of cell reprogramming. On the one hand, these data show that shear stress can influence the epigenetic state, on the other hand, excessive or inappropriate shear conditions can lead to adverse effects such as cell dysfunction or apoptosis (Yu et al., 2024), requiring careful optimization of microfluidic applications, which also applies to other devices.

In summary, while mechanical forces are vital for initiating cytoskeletal remodeling, there is no established pattern indicating which specific type of mechanical stimulation yields a particular reprogramming outcome.

### Biochemical agents and the cytoskeleton

In addition to physical factors, various biochemical agents can mimic cytoskeletal remodeling effects induced by biophysical stimuli, affecting cell shape and ultimately regulating lineage commitment (Table 1) (Figure 2). It has been well-established that changes in cell shape impact both proliferation and differentiation, largely through the modulation of Rho family GTPases and ROCK-mediated cytoskeletal tension (Sordella et al., 2003; McBeath et al., 2004). For instance, studies on mice deficient in p190-B RhoGAP, an inactivator of Rho, have shown decreased adipogenesis and increased myogenesis in embryonic fibroblasts (Sordella et al., 2003). Moreover, low ROCK activity, which corresponds with decreased myosin light chain phosphorylation, has been reported to influence MSCs' fate during differentiation (Bhadriraju et al., 2007).

**TABLE 1:** Effects of various biochemical agents on MSCs differentiation.

Small molecule	Effect on MSCs (cytoskeleton)	Differentiation outcome	References
Cytochalasin D	Inhibits F-actin polymerization	Adipogenesis	Pampanella et al. (2024)
Blebbistatin	Myosin II inhibitor, reduces intracellular tension	Adipogenesis decreased osteogenesis	Kilian et al. (2010)
Y-27632	ROCK inhibitor, reduces intracellular tension and myosin light chain phosphorylation	Adipogenesis, decreased osteogenesis	Kilian et al. (2010)
Nocodazole	Microtubule depolymerizing agent, enhances cell contractility	Osteogenesis	Kilian et al. (2010), Zhao et al. (2009)
Paclitaxel	ROCK inhibitor, induces aggregation and branching of actin filaments	Osteogenesis	McBride et al. (2008), Sen et al. (2017)
Latrunculin A, Latrunculin B	Inhibits actin polymerization, disrupts actin cytoskeleton	Adipogenesis, reduced osteogenesis	Lim et al. (2000), Sonowal et al. (2013)

MSCs, serve as a well-studied model for investigating how cytoskeletal changes affect fate determination. For example, the small molecule cytochalasin D, which inhibits F-actin polymerization, has been shown to favor adipogenic differentiation of MSCs. Recent reviews (Pampanella et al., 2024) have thoroughly examined cytochalasin’s effects on stem cell differentiation. Similarly, agents like blebbistatin (a myosin II inhibitor) and Y-27632 (a ROCK inhibitor) have been found to decrease osteogenesis while increasing adipocyte-like phenotypes in cells (Kilian et al., 2010). In contrast, nocodazole, a microtubule depolymerizing agent, enhances cell contractility, favoring osteogenic differentiation (Kilian et al., 2010).

Paclitaxel, another ROCK inhibitor, induces aggregation and branching of actin filaments, driving MSCs toward osteoblast differentiation. On the other hand, actin debranching tends to favor adipogenesis (McBride et al., 2008; Sen et al., 2017). These findings suggest that cell fate decisions are closely tied to actomyosin contractility, with the polymerization state of actin or tubulin serving as indicators of differentiation potential. A clear trend has emerged: increasing actin or microtubule polymerization promotes osteogenesis, whereas inhibiting polymerization supports adipogenesis. However, the decision for MSCs to commit to a specific lineage is far more complex than simply modulating actin or tubulin polymerization through chemical agents (Putra et al., 2023).

The effects of cytoskeletal remodeling vary significantly depending on the cell type. For -example, treating human pluripotent stem cells (hPSCs) with Latrunculin A during β-cell differentiation resulted in higher expression of NEUROG3, a marker of late pancreatic development (Hogrebe et al., 2020). In the same study, hPSCs treated with actin and microtubule polymerization inhibitors produced cells at different stages of differentiation within one population, with proportions influenced by the stages of actin and tubulin depolymerization (Hogrebe et al., 2020). In contrast, applying these agents to primary fibroblasts being reprogrammed into neurons yielded different outcomes. Specifically, blebbistatin and Y-27632 reduced intracellular tension and improved reprogramming efficiency, while nocodazole and cytochalasin D disrupted cell division, increased stress fibers, and ultimately reduced reprogramming efficiency (Soto et al., 2023). Reduction of cytoskeletal tension in primary fibroblasts using blebbistatin downregulates heterochromatin genes, decreases H3K27me3 and H3K9me3 marks, and promotes an open chromatin structure. This is reflected by increases in AcH3, H3K4me3, and H3K4me1 marks, as well as heightened HAT and HMT activity. Blebbistatin also reduces HDAC and HDM activity, ultimately promoting gene activation by enhancing histone acetylation and methylation (Soto et al., 2023). Notably, reducing actin cytoskeletal tension during the early stages of reprogramming facilitated a more open chromatin structure, decreased DNA methylation and heterochromatin marks, and increased euchromatin marks at neuronal gene promoters, enhancing reprogramming efficiency (Soto et al., 2023).

The underlying mechanisms of these effects are multifaceted. FA assembly is highly dependent on the force exerted between cells and their environment (Dumbauld et al., 2010). Inhibition of myosin II with blebbistatin significantly reduces vinculin localization to FA and decreases FA area on stiffer substrates (Zhou et al., 2017). Y-27632 treatment interferes with the recruitment of αTAT1, an acetyltransferase involved in microtubule acetylation, to FA and disrupts its interaction with talin (Seetharaman et al., 2022). Inhibitors of actin polymerization, such as cytochalasin D or latrunculin B, have been shown to activate protein kinase C alpha, promoting chondrogenic differentiation in chick embryo MSCs while favoring adipogenesis over osteogenesis in human bone marrow-derived MSCs (Lim et al., 2000; Sonowal et al., 2013). Both cytochalasin D and Latrunculin A also inhibit ERK and AKT activation, which are critical pathways for controlling cell proliferation, differentiation, and survival (Müller et al., 2013).

Moreover, disrupting the actin cytoskeleton with latrunculin A has been linked to reduced ERK1/2 phosphorylation and the subsequent decrease in cell proliferation in both human and murine rhabdomyosarcoma (Würtemberger et al., 2020). Nocodazole treatment has been shown to increase Ppar-γ expression and enhance BMP-2 promoter activity, leading to increased bone formation through the hedgehog signaling pathway (Zhao et al., 2009).

## Conclusion

In recent years, cytoskeletal components, particularly actin filaments and IFs, have emerged as critical players in cellular reprogramming and fate determination. This understanding highlights the cytoskeleton not just as a structural scaffold but as a dynamic mediator of biochemical and mechanical signals. Exploring the potential of targeting the cytoskeleton for improving reprogramming efficiency opens new avenues for regenerative medicine. Future research should focus on manipulating cytoskeletal regulators such as actin-binding proteins to enhance reprogramming outcomes. Additionally, further investigation into the integration of cytoskeletal dynamics with mechanotransduction pathways, including YAP/TAZ signaling, may lead to novel strategies for reprogramming cells into desired phenotypes more efficiently. Combining small molecules that target cytoskeletal remodeling with transcription factors could refine reprogramming protocols, paving the way for more predictable and efficient cell fate conversion. This area remains an exciting frontier for developing innovative therapeutic approaches in stem cell biology and regenerative medicine.

## References

[B1] Adams D. S. Keller R. Koehl M. A. (1990). The mechanics of notochord elongation, straightening and stiffening in the embryo of *Xenopus laevis* . Development 110, 115–130. 10.1242/dev.110.1.115 2081454

[B2] Akter R. Rivas D. Geneau G. Drissi H. Duque G. (2009). Effect of lamin A/C knockdown on osteoblast differentiation and function. J. Bone Min. Res. 24, 283–293. 10.1359/jbmr.081010 18847334

[B3] Alam S. G. Zhang Q. Prasad N. Li Y. Chamala S. Kuchibhotla R. (2016). The mammalian LINC complex regulates genome transcriptional responses to substrate rigidity. Sci. Rep. 6, 38063. 10.1038/srep38063 27905489 PMC5131312

[B4] Alfert A. Moreno N. Kerl K. (2019). The BAF complex in development and disease. Epigenetics Chromatin 12, 19. 10.1186/s13072-019-0264-y 30898143 PMC6427853

[B5] Almuzzaini B. Sarshad A. A. Farrants A.-K. Ö. Percipalle P. (2015). Nuclear myosin 1 contributes to a chromatin landscape compatible with RNA polymerase II transcription activation. BMC Biol. 13, 35. 10.1186/s12915-015-0147-z 26044184 PMC4486089

[B6] Almuzzaini B. Sarshad A. A. Rahmanto A. S. Hansson M. L. Von Euler A. Sangfelt O. (2016). In β-actin knockouts, epigenetic reprogramming and rDNA transcription inactivation lead to growth and proliferation defects. FASEB J. 30, 2860–2873. 10.1096/fj.201600280R 27127100

[B7] Arai R. En A. Takauji Y. Maki K. Miki K. Fujii M. (2019). Lamin B receptor (LBR) is involved in the induction of cellular senescence in human cells. Mech. Ageing Dev. 178, 25–32. 10.1016/j.mad.2019.01.001 30615890

[B8] Belay B. Mäntylä E. Maibohm C. Silvestre O. F. Hyttinen J. Nieder J. B. (2023). Substrate microtopographies induce cellular alignment and affect nuclear force transduction. J. Mech. Behav. Biomed. Mater. 146, 106069. 10.1016/j.jmbbm.2023.106069 37586175

[B9] Bera K. Kiepas A. Godet I. Li Y. Mehta P. Ifemembi B. (2022). Extracellular fluid viscosity enhances cell migration and cancer dissemination. Nature 611, 365–373. 10.1038/s41586-022-05394-6 36323783 PMC9646524

[B10] Bhadriraju K. Elliott J. T. Nguyen M. Plant A. L. (2007). Quantifying myosin light chain phosphorylation in single adherent cells with automated fluorescence microscopy. BMC Cell Biol. 8, 43. 10.1186/1471-2121-8-43 17941977 PMC2213650

[B11] Bian Q. Anderson E. C. Yang Q. Meyer B. J. (2020). Histone H3K9 methylation promotes formation of genome compartments in *Caenorhabditis elegans* via chromosome compaction and perinuclear anchoring. Proc. Natl. Acad. Sci. U. S. A. 117, 11459–11470. 10.1073/pnas.2002068117 32385148 PMC7261013

[B12] Bianchi A. Gervasi M. E. Bakin A. (2010). Role of β5-integrin in epithelial-mesenchymal transition in response to TGF-β. Cell Cycle 9, 1647–1659. 10.4161/cc.9.8.11517 20404485

[B13] Biferali B. Bianconi V. Perez D. F. Kronawitter S. P. Marullo F. Maggio R. (2021). Prdm16-mediated H3K9 methylation controls fibro-adipogenic progenitors identity during skeletal muscle repair. Sci. Adv. 7, eabd9371. 10.1126/sciadv.abd9371 34078594 PMC8172132

[B14] Blanchoin L. Boujemaa-Paterski R. Sykes C. Plastino J. (2014). Actin dynamics, architecture, and mechanics in cell motility. Physiol. Rev. 94, 235–263. 10.1152/physrev.00018.2013 24382887

[B15] Blanchoin L. Pollard T. D. (2002). Hydrolysis of ATP by polymerized actin depends on the bound divalent cation but not profilin. Biochemistry 41, 597–602. 10.1021/bi011214b 11781099

[B16] Bouzid T. Kim E. Riehl B. D. Esfahani A. M. Rosenbohm J. Yang R. (2019). The LINC complex, mechanotransduction, and mesenchymal stem cell function and fate. J. Biol. Eng. 13, 68. 10.1186/s13036-019-0197-9 31406505 PMC6686368

[B17] Boyle S. T. Kular J. Nobis M. Ruszkiewicz A. Timpson P. Samuel M. S. (2020). Acute compressive stress activates RHO/ROCK-mediated cellular processes. Small GTPases 11, 354–370. 10.1080/21541248.2017.1413496 29455593 PMC7549670

[B18] Brueckner L. Zhao P. A. van Schaik T. Leemans C. Sima J. Peric-Hupkes D. (2020). Local rewiring of genome-nuclear lamina interactions by transcription. EMBO J. 39, e103159. 10.15252/embj.2019103159 32080885 PMC7073462

[B19] Cabianca D. S. Muñoz-Jiménez C. Kalck V. Gaidatzis D. Padeken J. Seeber A. (2019). Active chromatin marks drive spatial sequestration of heterochromatin in *C. elegans* nuclei. Nature 569, 734–739. 10.1038/s41586-019-1243-y 31118512

[B20] Caliari S. R. Vega S. L. Kwon M. Soulas E. M. Burdick J. A. (2016). Dimensionality and spreading influence MSC YAP/TAZ signaling in hydrogel environments. Biomaterials 103, 314–323. 10.1016/j.biomaterials.2016.06.061 27429252 PMC4963302

[B21] Cao C. Xu Z. Liu Y. Cheng B. Xu F. (2023). Enhancement effects of extracellular fluid viscosity and matrix stiffness on cancer cell mechanosensing. Acta Mech. Sin. 39, 223238. 10.1007/s10409-023-23238-x

[B22] Carlier M. F. Laurent V. Santolini J. Melki R. Didry D. Xia G. X. (1997). Actin depolymerizing factor (ADF/cofilin) enhances the rate of filament turnover: implication in actin-based motility. J. Cell Biol. 136, 1307–1322. 10.1083/jcb.136.6.1307 9087445 PMC2132522

[B23] Chang L. Li M. Shao S. Li C. Ai S. Xue B. (2022). Nuclear peripheral chromatin-lamin B1 interaction is required for global integrity of chromatin architecture and dynamics in human cells. Protein Cell 13, 258–280. 10.1007/s13238-020-00794-8 33155082 PMC8934373

[B24] Chaudhuri O. Cooper-White J. Janmey P. A. Mooney D. J. Shenoy V. B. (2020). Effects of extracellular matrix viscoelasticity on cellular behaviour. Nature 584, 535–546. 10.1038/s41586-020-2612-2 32848221 PMC7676152

[B25] Chen X. Xu Z. Tang K. Hu G. Du P. Wang J. (2023). The mechanics of tumor cells dictate malignancy via cytoskeleton-mediated APC/Wnt/β-catenin signaling. Res. (Wash. D.C.) 6, 0224. 10.34133/research.0224 PMC10513157 37746658

[B26] Chen Y.-Q. Wu M.-C. Wei M.-T. Kuo J.-C. Yu H. W. Chiou A. (2024). High-viscosity driven modulation of biomechanical properties of human mesenchymal stem cells promotes osteogenic lineage. Mater Today Bio 26, 101058. 10.1016/j.mtbio.2024.101058 PMC11046220 38681057

[B27] Clapier C. R. Cairns B. R. (2009). The biology of chromatin remodeling complexes. Annu. Rev. Biochem. 78, 273–304. 10.1146/annurev.biochem.77.062706.153223 19355820

[B28] Crisp M. Liu Q. Roux K. Rattner J. B. Shanahan C. Burke B. (2006). Coupling of the nucleus and cytoplasm: role of the LINC complex. J. Cell Biol. 172, 41–53. 10.1083/jcb.200509124 16380439 PMC2063530

[B29] Dahl K. N. Kalinowski A. (2011). Nucleoskeleton mechanics at a glance. J. Cell Sci. 124, 675–678. 10.1242/jcs.069096 21321324 PMC3039014

[B30] Dash S. K. Sharma V. Verma R. S. Das S. K. (2020). Low intermittent flow promotes rat mesenchymal stem cell differentiation in logarithmic fluid shear device. Biomicrofluidics 14, 054107. 10.1063/5.0024437 33163135 PMC7595746

[B31] da Silva R. A. Fernandes C. J. da C. Feltran G. da S. Gomes A. M. de Camargo Andrade A. F. Andia D. C. (2019). Laminar shear stress-provoked cytoskeletal changes are mediated by epigenetic reprogramming of TIMP1 in human primary smooth muscle cells. J. Cell. Physiol. 234, 6382–6396. 10.1002/jcp.27374 30238981

[B32] Datta A. Deng S. Gopal V. Yap K. C.-H. Halim C. E. Lye M. L. (2021). Cytoskeletal dynamics in epithelial-mesenchymal transition: insights into therapeutic targets for cancer metastasis. Cancers 13, 1882. 10.3390/cancers13081882 33919917 PMC8070945

[B33] Desprat N. Supatto W. Pouille P.-A. Beaurepaire E. Farge E. (2008). Tissue deformation modulates twist expression to determine anterior midgut differentiation in Drosophila embryos. Dev. Cell 15, 470–477. 10.1016/j.devcel.2008.07.009 18804441

[B34] Diao Z. Ji Q. Wu Z. Zhang W. Cai Y. Wang Z. (2021). SIRT3 consolidates heterochromatin and counteracts senescence. Nucleic Acids Res. 49, 4203–4219. 10.1093/nar/gkab161 33706382 PMC8096253

[B35] Díaz-López A. Moreno-Bueno G. Cano A. (2014). Role of microRNA in epithelial to mesenchymal transition and metastasis and clinical perspectives. Cancer Manag. Res. 6, 205–216. 10.2147/CMAR.S38156 24812525 PMC4008290

[B36] Donnaloja F. Carnevali F. Jacchetti E. Raimondi M. T. (2020). Lamin A/C mechanotransduction in laminopathies. Cells 9, 1306. 10.3390/cells9051306 32456328 PMC7291067

[B37] Dugina V. B. Shagieva G. S. Kopnin P. B. (2022). Cytoplasmic beta and gamma actin isoforms reorganization and regulation in tumor cells in culture and tissue. Front. Pharmacol. 13, 895703. 10.3389/fphar.2022.895703 35721191 PMC9204531

[B38] Dumbauld D. W. Shin H. Gallant N. D. Michael K. E. Radhakrishna H. García A. J. (2010). Contractility modulates cell adhesion strengthening through focal adhesion kinase and assembly of vinculin-containing focal adhesions. J. Cell. Physiol. 223, 746–756. 10.1002/jcp.22084 20205236 PMC2874193

[B39] Dunlevy K. L. Medvedeva V. Wilson J. E. Hoque M. Pellegrin T. Maynard A. (2020). The PRR14 heterochromatin tether encodes modular domains that mediate and regulate nuclear lamina targeting. J. Cell Sci. 133, jcs240416. 10.1242/jcs.240416 32317397 PMC7272351

[B40] Dupont S. Morsut L. Aragona M. Enzo E. Giulitti S. Cordenonsi M. (2011). Role of YAP/TAZ in mechanotransduction. Nature 474, 179–183. 10.1038/nature10137 21654799

[B41] Elosegui-Artola A. Andreu I. Beedle A. E. M. Lezamiz A. Uroz M. Kosmalska A. J. (2017). Force triggers YAP nuclear entry by regulating transport across nuclear pores. Cell 171, 1397–1410. 10.1016/j.cell.2017.10.008 29107331

[B42] En A. Takauji Y. Miki K. Ayusawa D. Fujii M. (2020). Lamin B receptor plays a key role in cellular senescence induced by inhibition of the proteasome. FEBS Open Bio 10, 237–250. 10.1002/2211-5463.12775 PMC6996348 31825172

[B43] Engler A. J. Sen S. Sweeney H. L. Discher D. E. (2006). Matrix elasticity directs stem cell lineage specification. Cell 126, 677–689. 10.1016/j.cell.2006.06.044 16923388

[B44] Falk M. Feodorova Y. Naumova N. Imakaev M. Lajoie B. R. Leonhardt H. (2019). Heterochromatin drives compartmentalization of inverted and conventional nuclei. Nature 570, 395–399. 10.1038/s41586-019-1275-3 31168090 PMC7206897

[B45] Farrants A.-K. O. (2008). Chromatin remodelling and actin organisation. FEBS Lett. 582, 2041–2050. 10.1016/j.febslet.2008.04.032 18442483

[B46] Fletcher D. A. Mullins R. D. (2010). Cell mechanics and the cytoskeleton. Nature 463, 485–492. 10.1038/nature08908 20110992 PMC2851742

[B47] Fomproix N. Percipalle P. (2004). An actin-myosin complex on actively transcribing genes. Exp. Cell Res. 294, 140–148. 10.1016/j.yexcr.2003.10.028 14980509

[B48] Forsberg F. Brunet A. Ali T. M. L. Collas P. (2019). Interplay of lamin A and lamin B LADs on the radial positioning of chromatin. Nucleus 10, 7–20. 10.1080/19491034.2019.1570810 30663495 PMC6363278

[B49] Fu M. Hu Y. Lan T. Guan K.-L. Luo T. Luo M. (2022). The Hippo signalling pathway and its implications in human health and diseases. Signal Transduct. Target. Ther. 7, 376. 10.1038/s41392-022-01191-9 36347846 PMC9643504

[B50] Geiger T. Sabanay H. Kravchenko-Balasha N. Geiger B. Levitzki A. (2008). Anomalous features of EMT during keratinocyte transformation. PLoS One 3, e1574. 10.1371/journal.pone.0001574 18253510 PMC2215777

[B51] Gerardo H. Lima A. Carvalho J. Ramos J. R. D. Couceiro S. Travasso R. D. M. (2019). Soft culture substrates favor stem-like cellular phenotype and facilitate reprogramming of human mesenchymal stem/stromal cells (hMSCs) through mechanotransduction. Sci. Rep. 9, 9086. 10.1038/s41598-019-45352-3 31235788 PMC6591285

[B52] Gesson K. Rescheneder P. Skoruppa M. P. von Haeseler A. Dechat T. Foisner R. (2016). A-type lamins bind both hetero- and euchromatin, the latter being regulated by lamina-associated polypeptide 2 alpha. Genome Res. 26, 462–473. 10.1101/gr.196220.115 26798136 PMC4817770

[B53] Gonzalez-Molina J. Zhang X. Borghesan M. Mendonça da Silva J. Awan M. Fuller B. (2018). Extracellular fluid viscosity enhances liver cancer cell mechanosensing and migration. Biomaterials 177, 113–124. 10.1016/j.biomaterials.2018.05.058 29886384

[B54] Goodson H. V. Jonasson E. M. (2018). Microtubules and microtubule-associated proteins. Cold Spring Harb. Perspect. Biol. 10, a022608. 10.1101/cshperspect.a022608 29858272 PMC5983186

[B55] Guan J.-Q. Almo S. C. Reisler E. Chance M. R. (2003). Structural reorganization of proteins revealed by radiolysis and mass spectrometry: G-actin solution structure is divalent cation dependent. Biochemistry 42, 11992–12000. 10.1021/bi034914k 14556630

[B56] Gudimchuk N. B. McIntosh J. R. (2021). Regulation of microtubule dynamics, mechanics and function through the growing tip. Nat. Rev. Mol. Cell Biol. 22, 777–795. 10.1038/s41580-021-00399-x 34408299

[B57] Guelen L. Pagie L. Brasset E. Meuleman W. Faza M. B. Talhout W. (2008). Domain organization of human chromosomes revealed by mapping of nuclear lamina interactions. Nature 453, 948–951. 10.1038/nature06947 18463634

[B58] Guérette D. Khan P. A. Savard P. E. Vincent M. (2007). Molecular evolution of type VI intermediate filament proteins. BMC Evol. Biol. 7, 164. 10.1186/1471-2148-7-164 17854500 PMC2075511

[B59] Heo S.-J. Driscoll T. P. Thorpe S. D. Nerurkar N. L. Baker B. M. Yang M. T. (2016). Differentiation alters stem cell nuclear architecture, mechanics, and mechano-sensitivity. Elife 5, e18207. 10.7554/eLife.18207 27901466 PMC5148611

[B60] Herman A. B. Anerillas C. Harris S. C. Munk R. Martindale J. L. Yang X. (2021). Reduction of lamin B receptor levels by miR-340-5p disrupts chromatin, promotes cell senescence and enhances senolysis. Nucl. Acids Res. 49, 7389–7405. 10.1093/nar/gkab538 34181735 PMC8287953

[B61] Herrmann H. Aebi U. (2016). Intermediate filaments: structure and assembly. Cold Spring Harb. Perspect. Biol. 8, a018242. 10.1101/cshperspect.a018242 27803112 PMC5088526

[B62] Hirokawa N. Noda Y. Tanaka Y. Niwa S. (2009). Kinesin superfamily motor proteins and intracellular transport. Nat. Rev. Mol. Cell Biol. 10, 682–696. 10.1038/nrm2774 19773780

[B63] Ho J. W. K. Jung Y. L. Liu T. Alver B. H. Lee S. Ikegami K. (2014). Comparative analysis of metazoan chromatin organization. Nature 512, 449–452. 10.1038/nature13415 25164756 PMC4227084

[B64] Hofmann W. A. Stojiljkovic L. Fuchsova B. Vargas G. M. Mavrommatis E. Philimonenko V. (2004). Actin is part of pre-initiation complexes and is necessary for transcription by RNA polymerase II. Nat. Cell Biol. 6, 1094–1101. 10.1038/ncb1182 15502823

[B65] Hogan C. Varga-Weisz P. (2007). The regulation of ATP-dependent nucleosome remodelling factors. Mutat. Res. 618, 41–51. 10.1016/j.mrfmmm.2006.07.010 17306842

[B66] Hogrebe N. J. Augsornworawat P. Maxwell K. G. Velazco-Cruz L. Millman J. R. (2020). Targeting the cytoskeleton to direct pancreatic differentiation of human pluripotent stem cells. Nat. Biotechnol. 38, 460–470. 10.1038/s41587-020-0430-6 32094658 PMC7274216

[B67] Hohmann T. Dehghani F. (2019). The cytoskeleton-A complex interacting meshwork. Cells 8, 362. 10.3390/cells8040362 31003495 PMC6523135

[B68] Homayouni Moghadam F. Tayebi T. Moradi A. Nadri H. Barzegar K. Eslami G. (2014). Treatment with platelet lysate induces endothelial differentation of bone marrow mesenchymal stem cells under fluid shear stress. EXCLI J. 13, 638–649. 26417289 PMC4464185

[B69] Hsiao C. Lampe M. Nillasithanukroh S. Han W. Lian X. Palecek S. P. (2016). Human pluripotent stem cell culture density modulates YAP signaling. Biotechnol. J. 11, 662–675. 10.1002/biot.201500374 26766309 PMC4850094

[B70] Hu H. Ji Q. Song M. Ren J. Liu Z. Wang Z. (2020). ZKSCAN3 counteracts cellular senescence by stabilizing heterochromatin. Nucl. Acids Res. 48, 6001–6018. 10.1093/nar/gkaa425 32427330 PMC7293006

[B71] Hu P. Wu S. Hernandez N. (2004). A role for beta-actin in RNA polymerase III transcription. Genes Dev. 18, 3010–3015. 10.1101/gad.1250804 15574586 PMC535912

[B72] Hyman A. A. Salser S. Drechsel D. N. Unwin N. Mitchison T. J. (1992). Role of GTP hydrolysis in microtubule dynamics: information from a slowly hydrolyzable analogue, GMPCPP. Mol. Biol. Cell 3, 1155–1167. 10.1091/mbc.3.10.1155 1421572 PMC275679

[B73] Jain N. Iyer K. V. Kumar A. Shivashankar G. V. (2013). Cell geometric constraints induce modular gene-expression patterns via redistribution of HDAC3 regulated by actomyosin contractility. Proc. Natl. Acad. Sci. U. S. A. 110, 11349–11354. 10.1073/pnas.1300801110 23798429 PMC3710882

[B74] Janmey P. A. Fletcher D. A. Reinhart-King C. A. (2020). Stiffness sensing by cells. Physiol. Rev. 100, 695–724. 10.1152/physrev.00013.2019 31751165 PMC7276923

[B75] Kalluri R. Weinberg R. A. (2009). The basics of epithelial-mesenchymal transition. J. Clin. Invest. 119, 1420–1428. 10.1172/JCI39104 19487818 PMC2689101

[B76] Kelpsch D. J. Tootle T. L. (2018). Nuclear actin: from discovery to function. Anat. Rec. 301, 1999–2013. 10.1002/ar.23959 PMC6289869 30312531

[B77] Khatau S. B. Hale C. M. Stewart-Hutchinson P. J. Patel M. S. Stewart C. L. Searson P. C. (2009). A perinuclear actin cap regulates nuclear shape. Proc. Natl. Acad. Sci. U. S. A. 106, 19017–19022. 10.1073/pnas.0908686106 19850871 PMC2776434

[B78] Kilian K. A. Bugarija B. Lahn B. T. Mrksich M. (2010). Geometric cues for directing the differentiation of mesenchymal stem cells. Proc. Natl. Acad. Sci. U. S. A. 107, 4872–4877. 10.1073/pnas.0903269107 20194780 PMC2841932

[B79] Kim D.-H. Khatau S. B. Feng Y. Walcott S. Sun S. X. Longmore G. D. (2012). Actin cap associated focal adhesions and their distinct role in cellular mechanosensing. Sci. Rep. 2, 555. 10.1038/srep00555 22870384 PMC3412326

[B80] Kim D.-H. Wirtz D. (2013). Predicting how cells spread and migrate: focal adhesion size does matter. Cell adh. Migr. 7, 293–296. 10.4161/cam.24804 23628962 PMC3711996

[B81] King M. C. (2023). Dynamic regulation of LINC complex composition and function across tissues and contexts. FEBS Lett. 597, 2823–2832. 10.1002/1873-3468.14757 37846646

[B82] Kiss A. Fischer I. Kleele T. Misgeld T. Propst F. (2018). Neuronal growth cone size-dependent and -independent parameters of microtubule polymerization. Front. Cell. Neurosci. 12, 195. 10.3389/fncel.2018.00195 30065631 PMC6056669

[B83] Klages-Mundt N. L. Kumar A. Zhang Y. Kapoor P. Shen X. (2018). The nature of actin-family proteins in chromatin-modifying complexes. Front. Genet. 9, 398. 10.3389/fgene.2018.00398 30319687 PMC6167448

[B84] Kogut I. McCarthy S. M. Pavlova M. Astling D. P. Chen X. Jakimenko A. (2018). High-efficiency RNA-based reprogramming of human primary fibroblasts. Nat. Commun. 9, 745. 10.1038/s41467-018-03190-3 29467427 PMC5821705

[B85] Kukalev A. Nord Y. Palmberg C. Bergman T. Percipalle P. (2005). Actin and hnRNP U cooperate for productive transcription by RNA polymerase II. Nat. Struct. Mol. Biol. 12, 238–244. 10.1038/nsmb904 15711563

[B86] Kuo Y.-C. Chang T.-H. Hsu W.-T. Zhou J. Lee H.-H. Hui-Chun Ho J. (2015). Oscillatory shear stress mediates directional reorganization of actin cytoskeleton and alters differentiation propensity of mesenchymal stem cells. Stem Cells 33, 429–442. 10.1002/stem.1860 25302937

[B87] LeBlanc L. Kim M. Kambhampati A. Son A. J. Ramirez N. Kim J. (2022). β-catenin links cell seeding density to global gene expression during mouse embryonic stem cell differentiation. iScience 25, 103541. 10.1016/j.isci.2021.103541 34977504 PMC8689156

[B88] Legerstee K. Houtsmuller A. B. (2021). A layered view on focal adhesions. Biology 10, 1189. 10.3390/biology10111189 34827182 PMC8614905

[B89] Li Y. Zhong Z. Xu C. Wu X. Li J. Tao W. (2023). 3D micropattern force triggers YAP nuclear entry by transport across nuclear pores and modulates stem cells paracrine. Natl. Sci. Rev. 10, nwad165. 10.1093/nsr/nwad165 37457331 PMC10347367

[B90] Ligon L. A. Karki S. Tokito M. Holzbaur E. L. (2001). Dynein binds to beta-catenin and may tether microtubules at adherens junctions. Nat. Cell Biol. 3, 913–917. 10.1038/ncb1001-913 11584273

[B91] Lim Y. B. Kang S. S. Park T. K. Lee Y. S. Chun J. S. Sonn J. K. (2000). Disruption of actin cytoskeleton induces chondrogenesis of mesenchymal cells by activating protein kinase C-alpha signaling. Biochem. Biophys. Res. Commun. 273, 609–613. 10.1006/bbrc.2000.2987 10873653

[B92] Liu B. Wang J. Chan K. M. Tjia W. M. Deng W. Guan X. (2005). Genomic instability in laminopathy-based premature aging. Nat. Med. 11, 780–785. 10.1038/nm1266 15980864

[B93] Lou H.-Y. Zhao W. Li X. Duan L. Powers A. Akamatsu M. (2019). Membrane curvature underlies actin reorganization in response to nanoscale surface topography. Proc. Natl. Acad. Sci. U. S. A. 116, 23143–23151. 10.1073/pnas.1910166116 31591250 PMC6859365

[B94] Mandelkow E. Mandelkow E.-M. (2002). Kinesin motors and disease. Trends Cell Biol. 12, 585–591. 10.1016/s0962-8924(02)02400-5 12495847

[B95] Manzo S. G. Dauban L. van Steensel B. (2022). Lamina-associated domains: tethers and looseners. Curr. Opin. Cell Biol. 74, 80–87. 10.1016/j.ceb.2022.01.004 35189475

[B96] McBeath R. Pirone D. M. Nelson C. M. Bhadriraju K. Chen C. S. (2004). Cell shape, cytoskeletal tension, and RhoA regulate stem cell lineage commitment. Dev. Cell 6, 483–495. 10.1016/s1534-5807(04)00075-9 15068789

[B97] McBride S. H. Falls T. Knothe Tate M. L. (2008). Modulation of stem cell shape and fate B: mechanical modulation of cell shape and gene expression. Tissue Eng. Part A 14, 1573–1580. 10.1089/ten.tea.2008.0113 18774911

[B98] McIntosh J. R. (2016). Mitosis. Cold Spring Harb. Perspect. Biol. 8, a023218. 10.1101/cshperspect.a023218 27587616 PMC5008068

[B99] Mellad J. A. Warren D. T. Shanahan C. M. (2011). Nesprins LINC the nucleus and cytoskeleton. Curr. Opin. Cell Biol. 23, 47–54. 10.1016/j.ceb.2010.11.006 21177090

[B100] Melo-Fonseca F. Carvalho O. Gasik M. Miranda G. Silva F. S. (2023). Mechanical stimulation devices for mechanobiology studies: a market, literature, and patents review. Bio-Design Manuf. 6, 340–371. 10.1007/s42242-023-00232-8

[B101] Misu S. Takebayashi M. Miyamoto K. (2017). Nuclear actin in development and transcriptional reprogramming. Front. Genet. 8, 27. 10.3389/fgene.2017.00027 28326098 PMC5339334

[B102] Moujaber O. Stochaj U. (2020). The cytoskeleton as regulator of cell signaling pathways. Trends Biochem. Sci. 45, 96–107. 10.1016/j.tibs.2019.11.003 31812462

[B103] Müller P. Langenbach A. Kaminski A. Rychly J. (2013). Modulating the actin cytoskeleton affects mechanically induced signal transduction and differentiation in mesenchymal stem cells. PLoS One 8, e71283. 10.1371/journal.pone.0071283 23923061 PMC3726577

[B104] Na J. Yang Z. Shi Q. Li C. Liu Y. Song Y. (2024). Extracellular matrix stiffness as an energy metabolism regulator drives osteogenic differentiation in mesenchymal stem cells. Bioact. Mater. 35, 549–563. 10.1016/j.bioactmat.2024.02.003 38434800 PMC10909577

[B105] Nardone G. Oliver-De La Cruz J. Vrbsky J. Martini C. Pribyl J. Skládal P. (2017). YAP regulates cell mechanics by controlling focal adhesion assembly. Nat. Commun. 8, 15321. 10.1038/ncomms15321 28504269 PMC5440673

[B106] Naumanen P. Lappalainen P. Hotulainen P. (2008). Mechanisms of actin stress fibre assembly. J. Microsc. 231, 446–454. 10.1111/j.1365-2818.2008.02057.x 18755000

[B107] Nieto M. A. Huang R. Y.-J. Jackson R. A. Thiery J. P. (2016). EMT: 2016. Cell 166, 21–45. 10.1016/j.cell.2016.06.028 27368099

[B108] Nurmagambetova A. Mustyatsa V. Saidova A. Vorobjev I. (2023). Morphological and cytoskeleton changes in cells after EMT. Sci. Rep. 13, 22164. 10.1038/s41598-023-48279-y 38092761 PMC10719275

[B109] Obrdlik A. Percipalle P. (2011). The F-actin severing protein cofilin-1 is required for RNA polymerase II transcription elongation. Nucleus 2, 72–79. 10.4161/nucl.2.1.14508 21647301 PMC3104811

[B110] Oliver-De La Cruz J. Nardone G. Vrbsky J. Pompeiano A. Perestrelo A. R. Capradossi F. (2019). Substrate mechanics controls adipogenesis through YAP phosphorylation by dictating cell spreading. Biomaterials 205, 64–80. 10.1016/j.biomaterials.2019.03.009 30904599

[B111] Pampanella L. Petrocelli G. Abruzzo P. M. Zucchini C. Canaider S. Ventura C. (2024). Cytochalasins as modulators of stem cell differentiation. Cells 13, 400. 10.3390/cells13050400 38474364 PMC10931372

[B112] Pang Q. Y. Chiu Y.-C. Huang R. Y.-J. (2024). Regulating epithelial-mesenchymal plasticity from 3D genome organization. Commun. Biol. 7, 750. 10.1038/s42003-024-06441-w 38902393 PMC11190238

[B113] Parandakh A. Tafazzoli-Shadpour M. Khani M.-M. (2017). Stepwise morphological changes and cytoskeletal reorganization of human mesenchymal stem cells treated by short-time cyclic uniaxial stretch. Vitro Cell. Dev. Biol. Anim. 53, 547–553. 10.1007/s11626-017-0131-8 28205142

[B114] Park S.-M. Lee J.-H. Ahn K. S. Shim H. W. Yoon J.-Y. Hyun J. (2023). Cyclic stretch promotes cellular reprogramming process through cytoskeletal-nuclear mechano-coupling and epigenetic modification. Adv. Sci. 10, e2303395. 10.1002/advs.202303395 PMC10646259 37727069

[B115] Pascual-Reguant L. Blanco E. Galan S. Le Dily F. Cuartero Y. Serra-Bardenys G. (2018). Lamin B1 mapping reveals the existence of dynamic and functional euchromatin lamin B1 domains. Nat. Commun. 9, 3420. 10.1038/s41467-018-05912-z 30143639 PMC6109041

[B116] Pegoraro A. F. Janmey P. Weitz D. A. (2017). Mechanical properties of the cytoskeleton and cells. Cold Spring Harb. Perspect. Biol. 9, a022038. 10.1101/cshperspect.a022038 29092896 PMC5666633

[B117] Peng J.-M. Bera R. Chiou C.-Y. Yu M.-C. Chen T.-C. Chen C.-W. (2018). Actin cytoskeleton remodeling drives epithelial-mesenchymal transition for hepatoma invasion and metastasis in mice. Hepatology 67, 2226–2243. 10.1002/hep.29678 29171033

[B118] Pereira D. Richert A. Medjkane S. Hénon S. Weitzman J. B. (2020). Cell geometry and the cytoskeleton impact the nucleo-cytoplasmic localisation of the SMYD3 methyltransferase. Sci. Rep. 10, 20598. 10.1038/s41598-020-75833-9 33244033 PMC7691988

[B119] Peric-Hupkes D. Meuleman W. Pagie L. Bruggeman S. W. M. Solovei I. Brugman W. (2010). Molecular maps of the reorganization of genome-nuclear lamina interactions during differentiation. Mol. Cell 38, 603–613. 10.1016/j.molcel.2010.03.016 20513434 PMC5975946

[B120] Philimonenko V. V. Zhao J. Iben S. Dingová H. Kyselá K. Kahle M. (2004). Nuclear actin and myosin I are required for RNA polymerase I transcription. Nat. Cell Biol. 6, 1165–1172. 10.1038/ncb1190 15558034

[B121] Poleshko A. Mansfield K. M. Burlingame C. C. Andrake M. D. Shah N. R. Katz R. A. (2013). The human protein PRR14 tethers heterochromatin to the nuclear lamina during interphase and mitotic exit. Cell Rep. 5, 292–301. 10.1016/j.celrep.2013.09.024 24209742 PMC3867587

[B122] Poleshko A. Smith C. L. Nguyen S. C. Sivaramakrishnan P. Wong K. G. Murray J. I. (2019). H3K9me2 orchestrates inheritance of spatial positioning of peripheral heterochromatin through mitosis. Elife 8, e49278. 10.7554/eLife.49278 31573510 PMC6795522

[B123] Pollard T. D. (2016). Actin and actin-binding proteins. Cold Spring Harb. Perspect. Biol. 8, a018226. 10.1101/cshperspect.a018226 26988969 PMC4968159

[B124] Pollard T. D. Goldman R. D. (2018). Overview of the cytoskeleton from an evolutionary perspective. Cold Spring Harb. Perspect. Biol. 10, a030288. 10.1101/cshperspect.a030288 29967009 PMC6028065

[B125] Pongkitwitoon S. Uzer G. Rubin J. Judex S. (2016). Cytoskeletal configuration modulates mechanically induced changes in mesenchymal stem cell osteogenesis, morphology, and stiffness. Sci. Rep. 6, 34791. 10.1038/srep34791 27708389 PMC5052530

[B126] Putra V. D. L. Kilian K. A. Knothe Tate M. L. (2023). Biomechanical, biophysical and biochemical modulators of cytoskeletal remodelling and emergent stem cell lineage commitment. Commun. Biol. 6, 75. 10.1038/s42003-022-04320-w 36658332 PMC9852586

[B127] Rowley M. J. Corces V. G. (2018). Organizational principles of 3D genome architecture. Nat. Rev. Genet. 19, 789–800. 10.1038/s41576-018-0060-8 30367165 PMC6312108

[B128] Sankaran J. S. Sen B. Dudakovic A. Paradise C. R. Perdue T. Xie Z. (2020). Knockdown of formin mDia2 alters lamin B1 levels and increases osteogenesis in stem cells. Stem Cells 38, 102–117. 10.1002/stem.3098 31648392 PMC6993926

[B129] Sapra K. T. Qin Z. Dubrovsky-Gaupp A. Aebi U. Müller D. J. Buehler M. J. (2020). Nonlinear mechanics of lamin filaments and the meshwork topology build an emergent nuclear lamina. Nat. Commun. 11, 6205. 10.1038/s41467-020-20049-8 33277502 PMC7718915

[B130] Scaffidi P. Misteli T. (2008). Lamin A-dependent misregulation of adult stem cells associated with accelerated ageing. Nat. Cell Biol. 10, 452–459. 10.1038/ncb1708 18311132 PMC2396576

[B131] Schep R. Brinkman E. K. Leemans C. Vergara X. van der Weide R. H. Morris B. (2021). Impact of chromatin context on Cas9-induced DNA double-strand break repair pathway balance. Mol. Cell 81, 2216–2230.e10. 10.1016/j.molcel.2021.03.032 33848455 PMC8153251

[B132] Schmitter C. Di-Luoffo M. Guillermet-Guibert J. (2023). Transducing compressive forces into cellular outputs in cancer and beyond. Life Sci. Alliance 6, e202201862. 10.26508/lsa.202201862 37364915 PMC10292664

[B133] Schubert H. L. Wittmeyer J. Kasten M. M. Hinata K. Rawling D. C. Héroux A. (2013). Structure of an actin-related subcomplex of the SWI/SNF chromatin remodeler. Proc. Natl. Acad. Sci. U. S. A. 110, 3345–3350. 10.1073/pnas.1215379110 23401505 PMC3587198

[B134] Seetharaman S. Vianay B. Roca V. Farrugia A. J. De Pascalis C. Boëda B. (2022). Microtubules tune mechanosensitive cell responses. Nat. Mater. 21, 366–377. 10.1038/s41563-021-01108-x 34663953

[B135] Sen B. Uzer G. Samsonraj R. M. Xie Z. McGrath C. Styner M. (2017). Intranuclear actin structure modulates mesenchymal stem cell differentiation. Stem Cells 35, 1624–1635. 10.1002/stem.2617 28371128 PMC5534840

[B136] Sen B. Xie Z. Thomas M. D. Pattenden S. G. Howard S. McGrath C. (2024). Nuclear actin structure regulates chromatin accessibility. Nat. Commun. 15, 4095. 10.1038/s41467-024-48580-y 38750021 PMC11096319

[B137] Sen B. Xie Z. Uzer G. Thompson W. R. Styner M. Wu X. (2015). Intranuclear actin regulates osteogenesis. Stem Cells 33, 3065–3076. 10.1002/stem.2090 26140478 PMC4788101

[B138] Sgarzi M. Mazzeschi M. Santi S. Montacci E. Panciera T. Ferlizza E. (2023). Aberrant MET activation impairs perinuclear actin cap organization with YAP1 cytosolic relocation. Commun. Biol. 6, 1044. 10.1038/s42003-023-05411-y 37838732 PMC10576810

[B139] Sia J. Sun R. Chu J. Li S. (2016). Dynamic culture improves cell reprogramming efficiency. Biomaterials 92, 36–45. 10.1016/j.biomaterials.2016.03.033 27031931 PMC4983311

[B140] Sjöblom B. Salmazo A. Djinović-Carugo K. (2008). Alpha-actinin structure and regulation. Cell. Mol. Life Sci. 65, 2688–2701. 10.1007/s00018-008-8080-8 18488141 PMC11131806

[B141] Solovei I. Wang A. S. Thanisch K. Schmidt C. S. Krebs S. Zwerger M. (2013). LBR and lamin A/C sequentially tether peripheral heterochromatin and inversely regulate differentiation. Cell 152, 584–598. 10.1016/j.cell.2013.01.009 23374351

[B142] Song Y. Soto J. Chen B. Hoffman T. Zhao W. Zhu N. (2022). Transient nuclear deformation primes epigenetic state and promotes cell reprogramming. Nat. Mater. 21, 1191–1199. 10.1038/s41563-022-01312-3 35927431 PMC9529815

[B143] Sonowal H. Kumar A. Bhattacharyya J. Gogoi P. K. Jaganathan B. G. (2013). Inhibition of actin polymerization decreases osteogeneic differentiation of mesenchymal stem cells through p38 MAPK pathway. J. Biomed. Sci. 20, 71. 10.1186/1423-0127-20-71 24070328 PMC3849435

[B144] Sordella R. Jiang W. Chen G.-C. Curto M. Settleman J. (2003). Modulation of Rho GTPase signaling regulates a switch between adipogenesis and myogenesis. Cell 113, 147–158. 10.1016/s0092-8674(03)00271-x 12705864

[B145] Soto J. Song Y. Wu Y. Chen B. Park H. Akhtar N. (2023). Reduction of intracellular tension and cell adhesion promotes open chromatin structure and enhances cell reprogramming. Adv. Sci. 10, e2300152. 10.1002/advs.202300152 PMC10460843 37357983

[B146] Stowers R. S. Shcherbina A. Israeli J. Gruber J. J. Chang J. Nam S. (2019). Matrix stiffness induces a tumorigenic phenotype in mammary epithelium through changes in chromatin accessibility. Nat. Biomed. Eng. 3, 1009–1019. 10.1038/s41551-019-0420-5 31285581 PMC6899165

[B147] Su J.-H. Zheng P. Kinrot S. S. Bintu B. Zhuang X. (2020). Genome-scale imaging of the 3D organization and transcriptional activity of chromatin. Cell 182, 1641–1659. 10.1016/j.cell.2020.07.032 32822575 PMC7851072

[B148] Sulimenko V. Dráberová E. Dráber P. (2022). γ-Tubulin in microtubule nucleation and beyond. Front. Cell Dev. Biol. 10, 880761. 10.3389/fcell.2022.880761 36158181 PMC9503634

[B149] Swift J. Ivanovska I. L. Buxboim A. Harada T. Dingal P. C. D. P. Pinter J. (2013). Nuclear lamin-A scales with tissue stiffness and enhances matrix-directed differentiation. Science 341, 1240104. 10.1126/science.1240104 23990565 PMC3976548

[B150] Szeverenyi I. Cassidy A. J. Chung C. W. Lee B. T. K. Common J. E. A. Ogg S. C. (2008). The Human Intermediate Filament Database: comprehensive information on a gene family involved in many human diseases. Hum. Mutat. 29, 351–360. 10.1002/humu.20652 18033728

[B151] Tajik A. Zhang Y. Wei F. Sun J. Jia Q. Zhou W. (2016). Transcription upregulation via force-induced direct stretching of chromatin. Nat. Mater. 15, 1287–1296. 10.1038/nmat4729 27548707 PMC5121013

[B152] Takahashi K. Yamanaka S. (2006). Induction of pluripotent stem cells from mouse embryonic and adult fibroblast cultures by defined factors. Cell 126, 663–676. 10.1016/j.cell.2006.07.024 16904174

[B153] Titus M. A. (2018). Myosin-Driven intracellular transport. Cold Spring Harb. Perspect. Biol. 10, a021972. 10.1101/cshperspect.a021972 29496823 PMC5830894

[B154] Tsimbouri P. Gadegaard N. Burgess K. White K. Reynolds P. Herzyk P. (2014). Nanotopographical effects on mesenchymal stem cell morphology and phenotype. J. Cell. Biochem. 115, 380–390. 10.1002/jcb.24673 24123223

[B155] Ulianov S. V. Doronin S. A. Khrameeva E. E. Kos P. I. Luzhin A. V. Starikov S. S. (2019). Nuclear lamina integrity is required for proper spatial organization of chromatin in Drosophila. Nat. Commun. 10, 1176. 10.1038/s41467-019-09185-y 30862957 PMC6414625

[B156] Uzer G. Thompson W. R. Sen B. Xie Z. Yen S. S. Miller S. (2015). Cell mechanosensitivity to extremely low-magnitude signals is enabled by a LINCed nucleus. Stem Cells 33, 2063–2076. 10.1002/stem.2004 25787126 PMC4458857

[B157] van Steensel B. Belmont A. S. (2017). Lamina-associated domains: links with chromosome architecture, heterochromatin, and gene repression. Cell 169, 780–791. 10.1016/j.cell.2017.04.022 28525751 PMC5532494

[B158] Venit T. Xie X. Percipalle P. (2018). “15 - actin in the cell nucleus,” in Nuclear architecture and dynamics. Editors Lavelle C. Victor J.-M. (Boston: Academic Press), 345–367.

[B159] Vilar A. Hodgson-Garms M. Kusuma G. D. Donderwinkel I. Carthew J. Tan J. L. (2023). Substrate mechanical properties bias MSC paracrine activity and therapeutic potential. Acta Biomater. 168, 144–158. 10.1016/j.actbio.2023.06.041 37422008

[B160] Wang E. J.-Y. Chen I.-H. Kuo B. Y.-T. Yu C.-C. Lai M.-T. Lin J.-T. (2022). Alterations of cytoskeleton networks in cell fate determination and cancer development. Biomolecules 12, 1862. 10.3390/biom12121862 36551290 PMC9775460

[B161] Wang H. Yang Y. Liu J. Qian L. (2021). Direct cell reprogramming: approaches, mechanisms and progress. Nat. Rev. Mol. Cell Biol. 22, 410–424. 10.1038/s41580-021-00335-z 33619373 PMC8161510

[B162] Wang H. B. Dembo M. Hanks S. K. Wang Y. (2001). Focal adhesion kinase is involved in mechanosensing during fibroblast migration. Proc. Natl. Acad. Sci. U. S. A. 98, 11295–11300. 10.1073/pnas.201201198 11572981 PMC58723

[B163] Wang Y. Riechmann V. (2007). The role of the actomyosin cytoskeleton in coordination of tissue growth during Drosophila oogenesis. Curr. Biol. 17, 1349–1355. 10.1016/j.cub.2007.06.067 17656094

[B164] Wei M. Fan X. Ding M. Li R. Shao S. Hou Y. (2020). Nuclear actin regulates inducible transcription by enhancing RNA polymerase II clustering. Sci. Adv. 6, eaay6515. 10.1126/sciadv.aay6515 32494599 PMC7159918

[B165] Wong X. Hoskins V. E. Melendez-Perez A. J. Harr J. C. Gordon M. Reddy K. L. (2021). Lamin C is required to establish genome organization after mitosis. Genome Biol. 22, 305. 10.1186/s13059-021-02516-7 34775987 PMC8591896

[B166] Wozniak M. A. Chen C. S. (2009). Mechanotransduction in development: a growing role for contractility. Nat. Rev. Mol. Cell Biol. 10, 34–43. 10.1038/nrm2592 19197330 PMC2952188

[B167] Würtemberger J. Tchessalova D. Regina C. Bauer C. Schneider M. Wagers A. J. (2020). Growth inhibition associated with disruption of the actin cytoskeleton by Latrunculin A in rhabdomyosarcoma cells. PLoS One 15, e0238572. 10.1371/journal.pone.0238572 32898143 PMC7478754

[B168] Xiao Q. Hu X. Wei Z. Tam K. Y. (2016). Cytoskeleton molecular motors: structures and their functions in neuron. Int. J. Biol. Sci. 12, 1083–1092. 10.7150/ijbs.15633 27570482 PMC4997052

[B169] Xie N. Xiao C. Shu Q. Cheng B. Wang Z. Xue R. (2023). Cell response to mechanical microenvironment cues via Rho signaling: from mechanobiology to mechanomedicine. Acta Biomater. 159, 1–20. 10.1016/j.actbio.2023.01.039 36717048

[B170] Xie X. Almuzzaini B. Drou N. Kremb S. Yousif A. Farrants A.-K. Ö. (2018a). β-Actin-dependent global chromatin organization and gene expression programs control cellular identity. FASEB J. 32, 1296–1314. 10.1096/fj.201700753R 29101221

[B171] Xie X. Jankauskas R. Mazari A. M. A. Drou N. Percipalle P. (2018b). β-actin regulates a heterochromatin landscape essential for optimal induction of neuronal programs during direct reprograming. PLoS Genet. 14, e1007846. 10.1371/journal.pgen.1007846 30557298 PMC6312353

[B172] Xie X. Percipalle P. (2018). An actin-based nucleoskeleton involved in gene regulation and genome organization. Biochem. Biophys. Res. Commun. 506, 378–386. 10.1016/j.bbrc.2017.11.206 29203242

[B173] Ye J. Zhao J. Hoffmann-Rohrer U. Grummt I. (2008). Nuclear myosin I acts in concert with polymeric actin to drive RNA polymerase I transcription. Genes Dev. 22, 322–330. 10.1101/gad.455908 18230700 PMC2216692

[B174] Yeung T. Georges P. C. Flanagan L. A. Marg B. Ortiz M. Funaki M. (2005). Effects of substrate stiffness on cell morphology, cytoskeletal structure, and adhesion. Cell Motil. Cytoskelet. 60, 24–34. 10.1002/cm.20041 15573414

[B175] Yilmaz M. Christofori G. (2009). EMT, the cytoskeleton, and cancer cell invasion. Cancer Metastasis Rev. 28, 15–33. 10.1007/s10555-008-9169-0 19169796

[B176] Yu Z. Chen Y. Li J. Chen C. Lu H. Chen S. (2024). A tempo-spatial controllable microfluidic shear-stress generator for *in-vitro* mimicking of the thrombus. J. Nanobiotechnology 22, 187. 10.1186/s12951-024-02334-6 38632623 PMC11022418

[B177] Zhao M. Ko S.-Y. Liu J.-H. Chen D. Zhang J. Wang B. (2009). Inhibition of microtubule assembly in osteoblasts stimulates bone morphogenetic protein 2 expression and bone formation through transcription factor Gli2. Mol. Cell. Biol. 29, 1291–1305. 10.1128/MCB.01566-08 19103752 PMC2643819

[B178] Zhou D. W. Lee T. T. Weng S. Fu J. García A. J. (2017). Effects of substrate stiffness and actomyosin contractility on coupling between force transmission and vinculin-paxillin recruitment at single focal adhesions. Mol. Biol. Cell 28, 1901–1911. 10.1091/mbc.E17-02-0116 28468976 PMC5541841

[B179] Zhu X. Zeng X. Huang B. Hao S. (2004). Actin is closely associated with RNA polymerase II and involved in activation of gene transcription. Biochem. Biophys. Res. Commun. 321, 623–630. 10.1016/j.bbrc.2004.05.229 15358152

